# Global burden of stroke attributable to air pollution (1990–2021): An analysis of GBD 2021 data

**DOI:** 10.1097/MD.0000000000046419

**Published:** 2025-12-19

**Authors:** Changqiang Feng, Songxin Zhong, Chao Xiao, Rida Li, Xiaomin Feng, Shizhan Li, Zukun He, Yijiu Lu, Jianqing Zhu, Yanni Lin, Chao Qin

**Affiliations:** a Department of Neurology, The First Affiliated Hospital of Guangxi Medical University, Nanning, People’s Republic of China; b Department of Neurology, The First People’s Hospital of Yulin Affiliated to Guangxi Medical University, Yulin, People’s Republic of China; c Department of Neurology, The Affiliated Minzu Hospital of Guangxi Medical University, Nanning, People’s Republic of China.

**Keywords:** air pollution, Global Burden of Disease, public health intervention, stroke

## Abstract

This study aims to quantify the global burden of stroke attributable to air pollution and assess associated health disparities by region, gender, and socio-demographic index using 2021 Global Burden of Disease data. We conducted a systematic analysis of the 2021 Global Burden of Disease database, employing Joinpoint regression, decomposition analysis, and health inequality analysis to evaluate air pollution’s impact on stroke mortality and disability-adjusted life years. Analyses were stratified by nationality, region, gender, and socio-demographic index to identify health disparities. The study findings demonstrate a substantial increase in the global absolute burden of stroke attributable to air pollution from 1991 to 2021, with stroke-related mortality reaching 44,962,167 cases (95% uncertainty interval: 35,020,339–55,467,024) and disability-adjusted life years amounting to 44,962,167 person-years (95% uncertainty interval: 35,020,339–55,467,024). Notably, regional disparities in stroke burden have become increasingly pronounced. Furthermore, the disease burden was significantly higher among male patients compared to females, highlighting the critical role of gender disparities in the health impacts of stroke. Based on these findings, we recommend enhancing air pollution monitoring and control, strengthening health system resilience, and promoting targeted public health interventions. This study provides a scientific foundation for formulating prevention strategies against air pollution-related stroke and calls for strengthened global cooperation to address this critical public health challenge.

## 1. Introduction

As a global environmental issue, air pollution poses a significant threat to human health, with its potential impacts on cardiovascular and neurological diseases garnering increasing attention. According to the “2024 World Air Quality Report” published by IQAir, this study conducted an in-depth analysis of data from over 40,000 air quality monitoring stations across 8954 locations in 138 countries, regions, and territories, revealing the current state and trends of global air quality. The findings indicate that only 17% of cities worldwide met the World Health Organization (WHO) Particulate Matter 2.5 (PM2.5) guidelines in 2024, with over 99% of the global population residing in areas where air pollution levels exceeded the WHO Global Air Quality Guidelines limits. Notably, China’s national annual average PM2.5 concentration decreased from 32.5 µg/m³ in 2023 to 31 µg/m³ in 2024, which not only reflecting China’s efforts in air pollution control but also providing valuable insights for global air quality improvement.

Meanwhile, as the third leading cause of death globally, stroke poses multidimensional challenges to public health systems.^[1]^ According to the Global Burden of Disease (GBD) 2021 Stroke Burden Analysis published in ``The Lancet Neurology’’, stroke-related deaths reached 7.3 million in 2021. Approximately 30% of stroke survivors experience permanent motor or cognitive impairments, necessitating lifelong rehabilitation support. According to the GBD 2021 Stroke Burden Analysis published in *The Lancet Neurology*, stroke-related deaths reached 7.3 million in 2021. Approximately 30% of stroke survivors experience permanent motor or cognitive impairments, necessitating lifelong rehabilitation support.^[2]^ Research highlights that the top 5 risk factors for stroke globally in 2021 were high systolic blood pressure, ambient PM pollution, smoking, high levels of low-density lipoprotein cholesterol, and household air pollution.^[1]^ While progress has been made in controlling traditional risk factors such as hypertension and diabetes, the pathogenic effects of environmental exposures (e.g., PM2.5, ozone) remain inadequately quantified. Studies indicate that air pollution exposure is associated with 20% of the global stroke burden and contributes to approximately 16% of stroke-related deaths.^[3]^ Notably, exposure to PM is a significant predictor of stroke incidence and mortality.^[4]^

Recent years have witnessed groundbreaking advancements in understanding the mechanisms linking air pollution to stroke. A 2024 cohort study demonstrated that PM2.5 directly increases stroke risk through pathways such as inducing systemic inflammatory responses (IL-6 increased by 42%), endothelial dysfunction (Flow-Mediated Dilation reduced by 19%), and blood–brain barrier disruption (S100β increased by 31%). Notably, the population attributable fraction for PM2.5 reached 24.3% in industrialized regions of Asia.^[5,6]^ However, current research lacks systematic analyses of the spatiotemporal heterogeneity, dose–response relationships, and intervention priorities regarding the association between air pollutants and stroke burden. There is an urgent need for multidimensional analyses based on the GBD database to provide robust evidence for public health interventions.

This study aims to conduct a comprehensive analysis of the global burden of stroke attributable to air pollution using data from the GBD 2021. Employing advanced methodologies, including Joinpoint regression, decomposition analysis, and Frontier analysis, we systematically tracked the impact of air pollution on stroke mortality and disability-adjusted life years. The burden was further stratified by country, region, sex, and socio-demographic index (SDI) to identify high-risk populations and areas for targeted interventions. Through this research, we seek to provide robust scientific evidence for the formulation of global stroke prevention and control strategies, while also advancing the understanding of the intricate relationship between air pollution and stroke.

## 2. Methods

### 2.1. Data origins and research entities

Stroke was defined according to WHO criteria as rapidly developing clinical signs of focal (or less commonly global) disturbance of cerebral function lasting more than 24 hours or leading to death with no apparent cause other than that of vascular origin. Cases of transient ischemic attack were not included. In this study, we utilized the GBD 2021 database as the primary data source. This comprehensive database provides detailed epidemiological estimates of 371 diseases and injuries across 21 GBD regions and 204 countries and territories from 1990 to 2021.^[7]^ The data are publicly accessible through the Global Health Data Exchange (https://ghdx.healthdata.org/gbd-2021/sources), ensuring the transparency and reproducibility of our research. According to the International Classification of Diseases 10th editions, ischemic stroke is represented by codes G45-G46.8, I60-I62, I62.9-I64, I64.1, I65-I69.998. Our analysis within the GBD framework specifically focused on the relationship between air pollution and the burden of stroke. We meticulously calculated the contribution of air pollution to the stroke disease burden. Accounting for the influence of varying population age structures on our findings, we employed the age-standardized rate (ASR) to standardize stroke mortality and disability-adjusted life years (DALYs). This methodological approach ensures that our results accurately reflect the true trends in disease burden, independent of population structure disparities.

### 2.2. Indicators

We utilized 2 core metrics to evaluate the disease burden of stroke in essential healthcare services: mortality rate and disability-adjusted life year rate. To ensure the accuracy and comparability of our analytical results, we standardized the data using the ASR, thereby eliminating the potential influence of age structure on disease burden assessment.^[7]^ The DALYs is a comprehensive measure of health loss, calculated by integrating years of life lost and years lived with disability due to premature death. This metric not only captures the impact of diseases on the quantity of life but also quantifies their detrimental effects on the quality of life, providing a robust and multidimensional framework for disease burden research. Furthermore, this study incorporated the SDI as a composite indicator to assess the developmental status of countries or regions. The SDI is constructed by synthesizing several critical socio-economic factors, including the total fertility rate for women under 25, the average education level of the population aged 15 and above, and per capita income. The SDI ranges from 0 to 1, with higher values indicating a higher level of socio-economic development. By integrating the SDI into our analysis, we were able to explore more deeply the association between socio-economic development levels and disease burden of stroke. This approach provides a scientific foundation for the formulation of targeted public health intervention strategies, enhancing our understanding of the complex interplay between socio-economic factors and health outcomes.

### 2.3. Grouped by region

This study conducts a systematic analysis of air pollution and stroke-related data across 204 countries and 21 GBD regions in the GBD database. These regions are comprised of geographically proximate countries with similar epidemiological profiles, facilitating a more representative assessment of regional disease burdens. To explore the association between stroke burden and socio-economic development, the SDI was calculated for each country. Based on SDI values, the 204 countries and territories were categorized into 5 distinct groups: high SDI (>0.81), high-medium SDI (0.7–0.81), medium SDI (0.61–0.70), low-medium SDI (0.46–0.61), and low SDI (<0.46). This stratification method not only elucidates the distribution patterns of stroke burden across varying socio-economic development levels but also provides critical insights into regional disparities and underlying drivers in disease burden. Consequently, it offers a theoretical foundation for developing targeted public health intervention strategies and optimizing resource allocation.

### 2.4. Risk factors

The study findings reveal a significant increase in global stroke-related DALYs attributable to 23 risk factors, rising from 100 million years of health loss in 1990 to 135 million years in 2021, highlighting the persistent upward trend in the burden of stroke.^[1]^ At the global level, the 5 major risk factors for stroke are, in descending order: high systolic blood pressure, ambient PM pollution, smoking, high levels of low-density lipoprotein cholesterol, and household air pollution.^[1]^ The distribution of these risk factors exhibits significant heterogeneity across different age groups, genders, and regions, indicating the necessity for tailored stroke prevention strategies based on population characteristics. Taking subarachnoid hemorrhage (SAH) as an example, a severe subtype of stroke, its primary risk factor is ambient PM pollution, accounting for 14% of SAH-related deaths and disabilities, with an impact comparable to smoking. This study focuses on the impact of air pollution risk factors on the burden of stroke across different regions, countries, and genders, aiming to provide a scientific basis for the development of regionalized and precise stroke prevention and intervention strategies, while also offering data support for the optimization of global public health policies.

### 2.5. Statistical analysis

This study systematically compared the age-standardized mortality rate (ASMR) and DALYs rate of stroke patients across different countries, regions and genders, providing an in-depth exploration of the epidemiological characteristics and spatiotemporal trends of stroke. The ASR was calculated per 100,000 population using the following formula: $Age\text{-}standardised\;\;\;rates\;\;\;=\;\;\;\frac{\overset{A}{\underset{i=1}{\sum }}a_{i}w_{i}}{\overset{A}{\underset{i=1}{\sum }}w_{i}}\;\;\;\times 1$ 100,000, where ai and wi denote age-specific rates and the number of persons (or weight) in the same age subgroup of the chosen reference standard population (where i denotes the ith age class), respectively. This method ensures comparability across populations with different demographic structures. To further analyze the temporal trends of ASR, the average annual percentage change (AAPC) was calculated. The formula is as follows: Y = α+ βX + e, where Y represents the natural logarithm of ASR, X denotes the calendar year, αis the intercept, β is the slope or trend, and e is the error term. The AAPC was calculated as: 100 × [exp(β) ‐ 1], representing the annual percentage change. A linear regression model was employed to estimate the 95% confidence interval (CI) of the AAPC. Two distinct uncertainty intervals were employed according to analytical approaches: 95% CI for classical statistical inference using raw observational data; 95% uncertainty intervals (95% UI) for modeled estimates incorporating multiple data sources (e.g., GBD hierarchical models) or Bayesian analyses. This differentiation follows IHME technical standards to properly account for different uncertainty sources. If both the EAPC and the lower limit of its 95% CI were positive, the ASR was considered to exhibit an increasing trend. Conversely, if both the EAPC and the upper limit of its 95% CI were negative, the ASR was considered to exhibit a decreasing trend. If neither condition was met, the age-standardized rate was deemed stable. Jointpoint regression was employed to analyze the temporal trends of disease burden, aiming to identify significant change points in indicators such as mortality and DALYs. Initially, relevant data from the GBD study were included and subjected to quality control to ensure data integrity and accuracy. Subsequently, the Jointpoint software (Version 5.3, joinpoint@imsweb.com) was utilized for analysis. Our decomposition analyses draw from methods developed by Das Gupta to provide a computationally tractable solution for isolating drivers of burden changes whereby all combinations of possible pathways are averaged across factors. In health inequality analysis, the slope index of inequality and concentration index are employed to assess disparities in disease burden across socioeconomic statuses. For detailed methodological descriptions, please refer to the File S1, Supplemental Digital Content, https://links.lww.com/MD/Q927. Through these methods, this study not only revealed the global distribution characteristics of the stroke burden but also quantified its temporal trends, providing a scientific basis for the development of targeted prevention and intervention strategies.

## 3. Results

### 3.1. Global level

**Figure 1.**
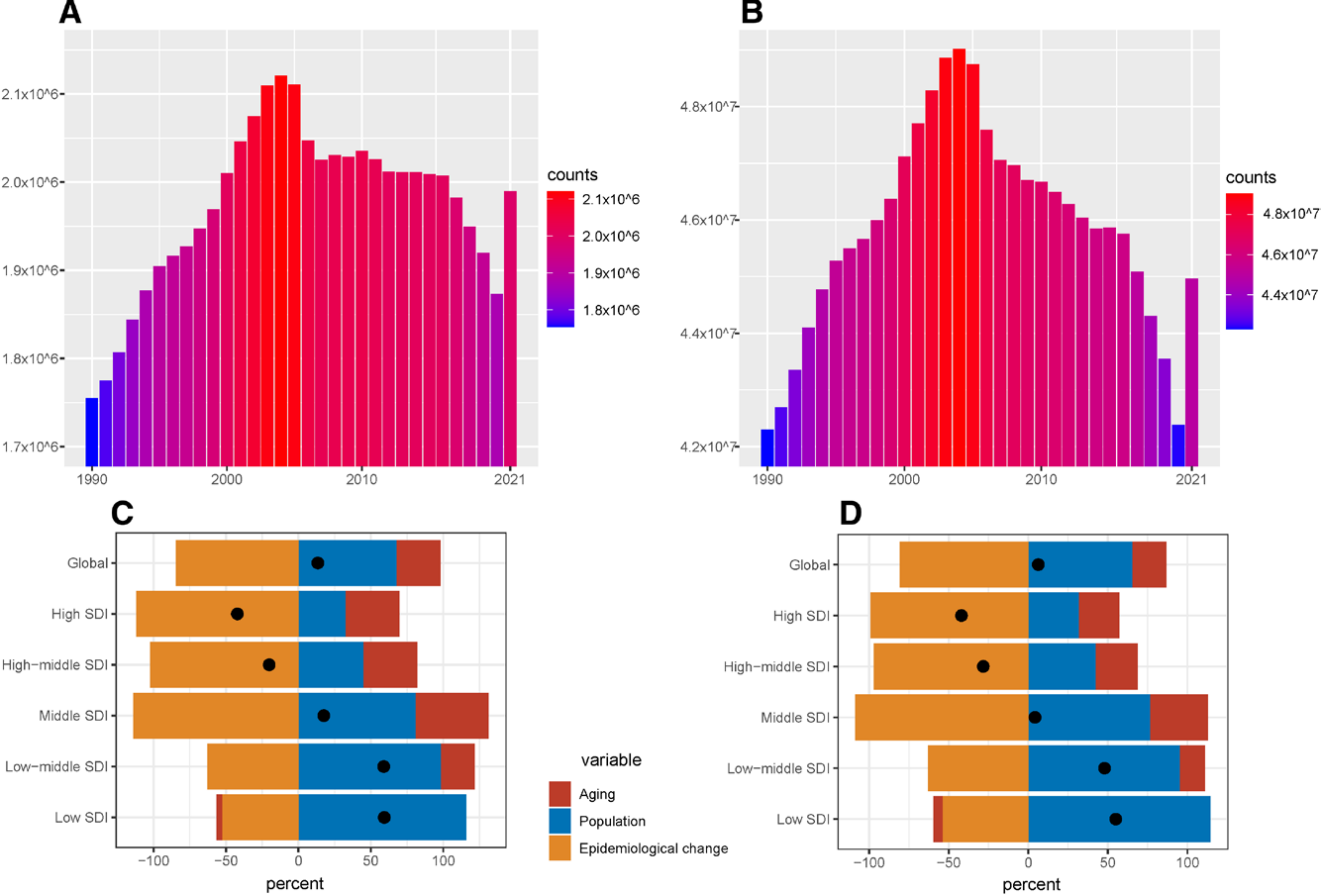
Trends of global disease burden of stroke attributable to air pollution from 1990 to 2021. (A) Mortality attributable to air pollution. (B) DALYs attributable to air pollution. (C) Percent change of mortality attributable to air pollution. (D) Percent change of DALYs attributable to air pollution. DALYs = disability-adjusted life year rates, SDI = socio-demographic index.

As shown in Table 1 and Figure 1, the disease burden of stroke attributable to air pollution remained significant in 2021, with global deaths reaching 1,989,686 cases (95% UI: 1,530,479–2,493,238), representing a 13.4% increase compared to 1,755,017 cases (95% UI: 1,434,139–2,094,574) in 1990. However, the ASMR exhibited a significant downward trend, decreasing from 48.86 per 100,000 population (95% UI: 39.69–58.76) in 1990 to 23.74 per 100,000 population (95% UI: 18.26–29.8) in 2021, with an AAPC of −2.43 (95% CI: −2.49 to −2.37). These findings indicate that, although the absolute number of deaths increased, the mortality rate of stroke significantly declined after accounting for population growth and changes in age structure. Furthermore, as presented in Table 1, the DALYs attributable to air pollution increased from 42,304,118 person-years (95% UI: 34,553,910–49,981,910) in 1990 to 44,962,167 person-years (95% UI: 35,020,339–55,467,024) in 2021. Nevertheless, the age-standardized disability-adjusted life year rate (ASDR) also demonstrated a declining trend, decreasing from 1073.52 per 100,000 population (95% UI: 877.41–1276.32) in 1990 to 523.3 per 100,000 population (95% UI: 407.96–645.58) in 2021, with an AAPC of −2.42 (95% CI: −2.47 to −2.36). The decomposition analysis in Figure 1 revealed that global population aging and population growth substantially contributed to the increased burden of stroke, while epidemiological changes mitigated the burden of stroke. These results underscore the complex interplay between demographic shifts and epidemiological factors in shaping the global burden of stroke attributable to air pollution.

**Table 1:** The mortality cases and DALYs of stroke due to air pollution in 1990 and 2021.

	1990		2021		AAPC (95%CI)
	Number (95% UI)	Age-standardized rate/(10^6^) (95% UI)	Number (95% UI)	Age-standardized rate/(10^6^) (95% UI)	
Mortality					
Global	1,755,017.25 (1,434,138.6–2,094,574.28)	48.86 (39.69–58.76)	1,989,686.32 (1,530,479.07–2,493,237.87)	23.74 (18.26–29.8)	‐2.43 (-2.49 to -2.37)
Male	865,991.61 (696,794.86–1,043,249.92)	54.98 (44.18–66.38)	1,055,764.05 (798,130.97–1,328,422.25)	28.31 (21.42–35.65)	-2.17 (-2.27 to -2.12)
Female	889,025.64 (713,428.28–1,079,934.85)	44.05 (35.34–53.63)	933,922.27 (726,991.02–1,174,376.35)	20.06 (15.62–25.22)	-2.64 (-2.69 to -2.58)
High SDI	141,013.07 (96,959.75–196,349.16)	12.68 (8.68–17.67)	81,577.09 (60,005.23–107,352.29)	3.46 (2.59–4.51)	-4.14 (-4.21 to -4.09)
High-middle SDI	487,241.13 (377,423.01–608,683.31)	55.94 (42.8–70.51)	388,996.79 (292,154.97–512,267.73)	20.01 (15.04–26.35)	-3.49 (-3.57 to -3.38)
Middle SDI	653,037.38 (533,201.82–791,568.52)	77.73 (63.29–94.43)	767,478.72 (568,986.82–1,008,332.62)	32.11 (23.83–42.15)	-2.96 (-3.06 to -2.88)
Low-middle SDI	330,493.89 (269,286.1–389,627.09)	63.85 (52.03–75.39)	525,073.74 (411,295.13–629,590.89)	41.42 (32.35–49.81)	-1.4 (-1.47 to -1.33)
Low SDI	141,329.84 (114,505.34–167,203.68)	75.55 (61.78–89.16)	225,105.31 (180,623.01–266,855.14)	53.87 (43.37–63.85)	-1.08 (-1.11 to -1.04)
DALYs					
Global	42,304,117.5 (34,553,909.64–49,981,909.54)	1073.52 (877.41–1276.32)	44,962,166.97 (35,020,338.76–55,467,023.53)	523.3 (407.96–645.58)	-2.42 (-2.47 to -2.36)
Male	22,042,113.99 (17,840,356.68–26,509,029.51)	1211.35 (978.24–1459.82)	24,804,489.97 (18,892,806.31–31,076,582.44)	619.37 (471.91–777.85)	-2.21 (-2.31 to -2.15)
Female	20,262,003.51 (16,469,195.78–24,428,886.13)	955.8 (776.39–1152.5)	20,157,677.01 (15,842,114.84–25,207,194.65)	439 (345.08–549.03)	-2.6 (-2.66 to -2.54)
High SDI	2,802,117.29 (1,970,060.92–3,869,337.02)	254.27 (178.36–350.52)	1,625,946.6 (1,232,130.37–2,082,328.67)	80.32 (61.68–102.39)	-3.68 (-3.74 to -3.64)
High-middle SDI	10,874,231.62 (8,518,190.42–13,473,283.43)	1133.11 (886.3–1405.11)	7,801,888.17 (5,931,357.33–10,188,263.45)	398.87 (303.25–520.76)	-3.36 (-3.48 to -3.25)
Middle SDI	16,139,883.95 (13,229,208.13–19,323,108.98)	1617.52 (1322.7–1942.01)	16,823,973.99 (12,428,844.31–21,838,218.68)	645.43 (478.24–838.92)	-2.97 (-3.14 to -2.89)
Low-middle SDI	8,677,233 (7,121,160.17–10,185,553.84)	1422.35 (1162.67–1675.4)	12,835,904.8 (10,109,818.69–15,248,022.76)	899.28 (707.45–1071.12)	-1.49 (-1.54 to -1.42)
Low SDI	3,768,690.01 (3,076,522.18–4,476,852.89)	1680.76 (1375.99–1984.48)	5,841,672.12 (4,664,357.38–6,927,648.54)	1162.18 (934.76–1380.38)	-1.2 (-1.22 to -1.17)

AAPC = average annual percentage change, CI = confidence interval, SDI = sociodemographic index, UI = uncertainty interval.

### 3.2. Regional level

**Figure 2.**
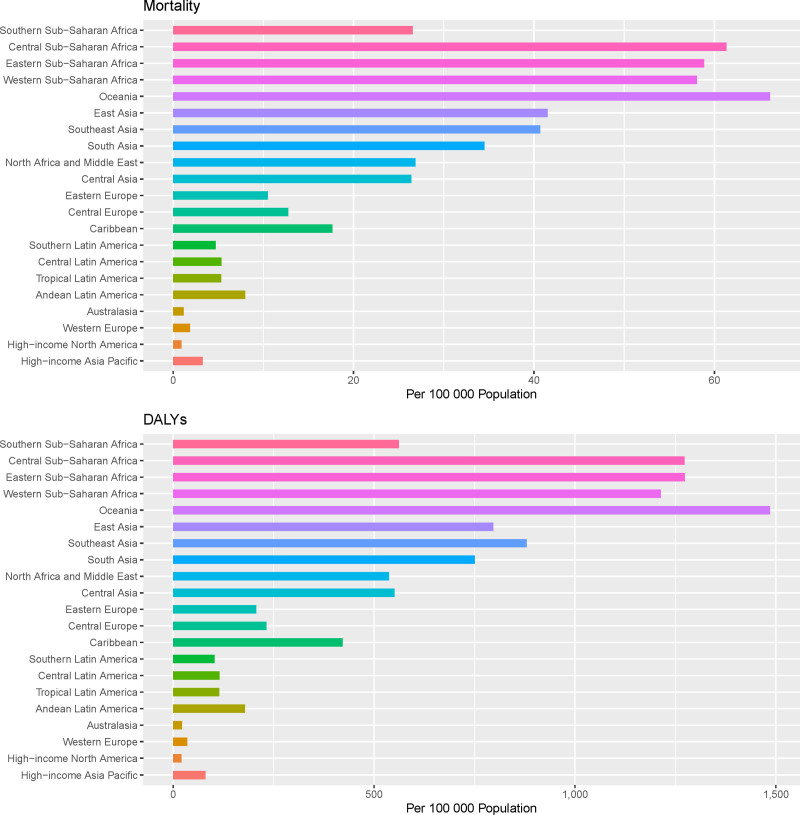
The age-standardized rate of mortality and DALYs (per 100,000) of stroke attributable to air pollution in 2021 across 21 GBD regions. DALYs = disability-adjusted life year rates, GBD = the Global Burden of Disease.

**Figure 3.**
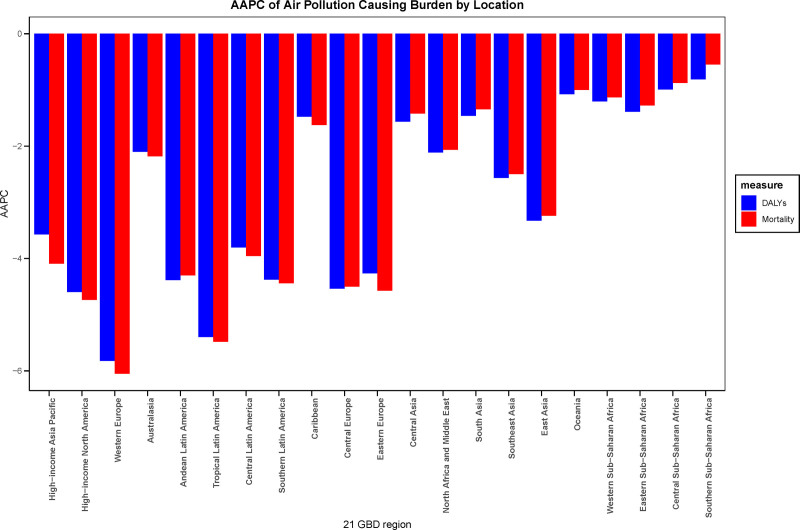
Global trends in age-standardized mortality and DALYs (1990–2021) across 21 GBD regions. AAPC = average annual percentage change, DALYs = disability-adjusted life year rates, GBD = the Global Burden of Disease.

This study systematically evaluated the impact of air pollution on stroke burden across 21 regions globally, based on the GBD regional classification standards. The results revealed significant regional disparities in stroke burden attributable to air pollution. In 1990, the 3 regions with the highest number of stroke deaths attributable to air pollution were East Asia (745,481 deaths, 95% UI: 590,872–913,566), South Asia (250,053 deaths, 95% UI: 198,667–297,584), and Southeast Asia (188,257 deaths, 95% UI: 148,223–226,024). Similarly, the highest DALYs were observed in East Asia (17,984,929 person-years, 95% CI: 14,430,400–22,079,589), South Asia (6698,029 person-years, 95% UI: 847,160–1260,230), and Southeast Asia (49,406,423 person-years, 95% CI: 39,435,88–58,664,77). By 2021, the regions with the highest stroke deaths remained East Asia (815,599 deaths, 95% UI: 593,056–1078,387), South Asia (4,499,966 deaths, 95% UI: 5,379,939–7,941,527), and Southeast Asia (230,470 deaths, 95% UI: 160,755–307,503). The DALYs ranking was consistent with 1990, with East Asia (16,774,343 person-years, 95% CI: 12,321,391–22,074,556), South Asia (6,698,029 person-years, 95% UI: 847,160–1,260,230), and Southeast Asia (5,692,351 person-years, 95% CI: 3,958,956–7,553,311) leading the list. After adjusting for population size and aging factors, East Asia had the highest ASMR in 1990 (113.43 per 100,000, 95% UI: 90.37–138.12), followed by Oceania (90.59 per 100,000, 95% UI: 68.32–115.86) and Southeast Asia (87.72 per 100,000, 95% UI: 69.02–105.65). By 2021, the top 3 regions for age-standardized mortality were Oceania (66.15 per 100,000, 95% UI: 48.16–86.24), Central Sub-Saharan Africa (61.34 per 100,000, 95% UI: 44.12–83.87), and Eastern Sub-Saharan Africa (58.85 per 100,000, 95% UI: 46.7–71.65), as illustrated in Figure 2. In terms of age-standardized DALYs, the top 3 regions in 1990 were East Asia (2232.68 per 100,000, 95% UI: 1783.44–2718.40), Oceania (2056.51 per 100,000, 95% UI: 1537.83–2653.98), and Eastern Sub-Saharan Africa (1966.06 per 100,000, 95% UI: 563.10–1349.13). By 2021, the leading regions were Oceania (1485.18 per 100,000, 95% CI: 1081.84–1941.14), Eastern Sub-Saharan Africa (1273.51 per 100,000, 95% CI: 1009.75–1537.19), and Central Sub-Saharan Africa (1272.78 per 100,000, 95% CI: 919.31–1708.91). Globally, age-standardized mortality and DALYs showed a declining trend across all 21 regions, as depicted in Figure 3, Western Europe exhibited the most significant decline, with an AAPC of −6.05 (95% CI: −6.17 to −5.95) for age-standardized mortality and −5.82 (95% CI: −5.92 to −5.74) for DALYs. Latin America followed closely, with an AAPC of −5.48 (95% CI: −5.59 to −5.40) for mortality and −5.40 (95% CI: −5.51 to −5.31) for DALYs. In contrast, Southern Sub-Saharan Africa experienced the smallest decline, with an AAPC of −0.55 (95% CI: −5.59 to −5.40) for mortality and −0.81 (95% CI: −0.62 to −0.47) for DALYs.

### 3.3. Burden of ambient air pollution based on SDI

**Figure 4.**
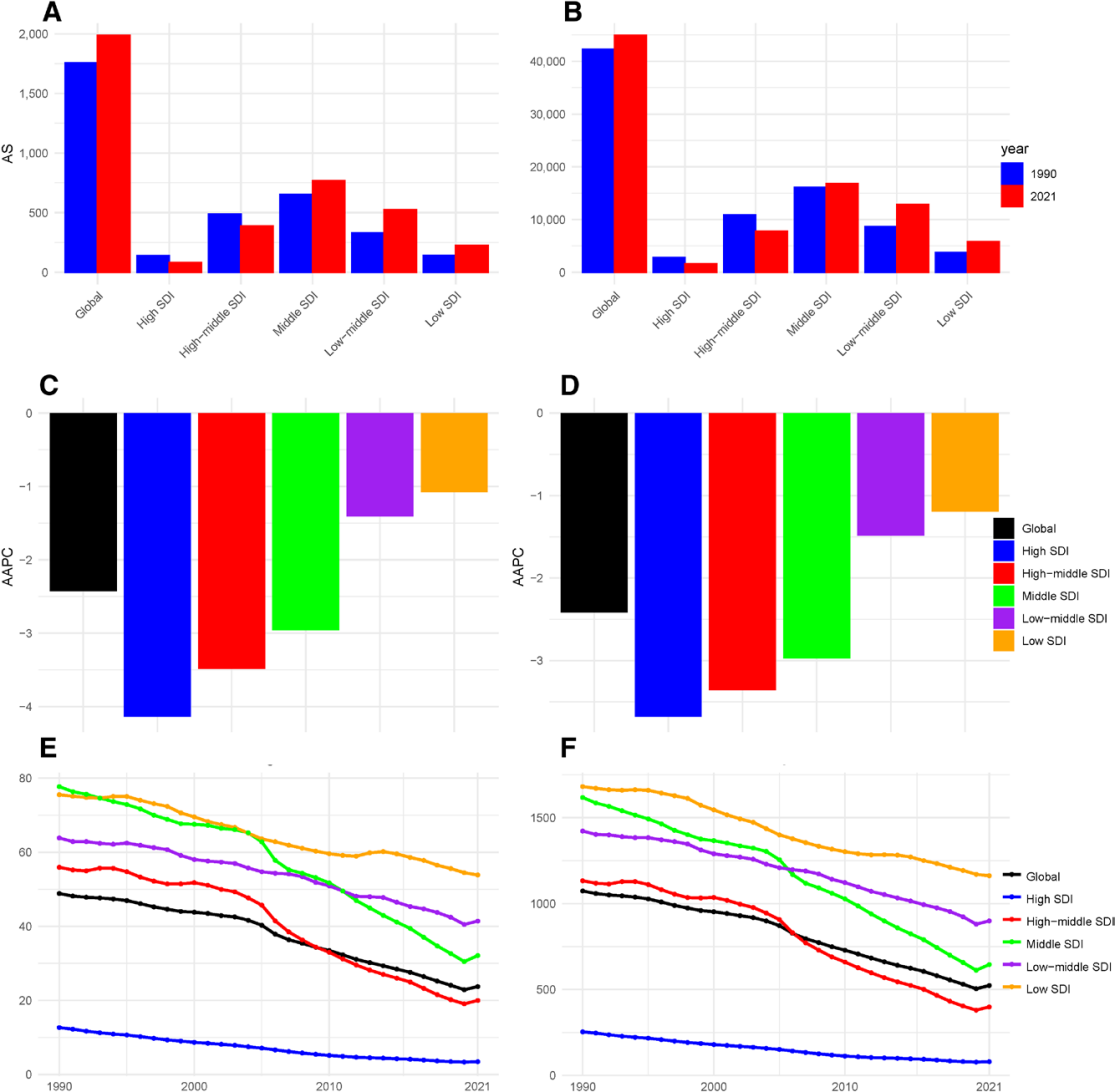
Age-standardized burden rate attributable to air pollution across regions ranked by SDI in 1990 and 2021. (A) Age-standardized mortality rate attributable to air pollution. (B) Age-standardized disability-adjusted life year rate attributable to air pollution. (C) AAPC of mortality attributable to air pollution. (D) AAPC of DALYs attributable to air pollution. (E) Trends in ASMR attributable to air pollution. (F) Trends in DALYs attributable to air pollution. AAPC = average annual percentage change, ASMR = age-standardized mortality rate, DALYs = disability-adjusted life year rates, SDI = sociodemographic index.

**Figure 5.**
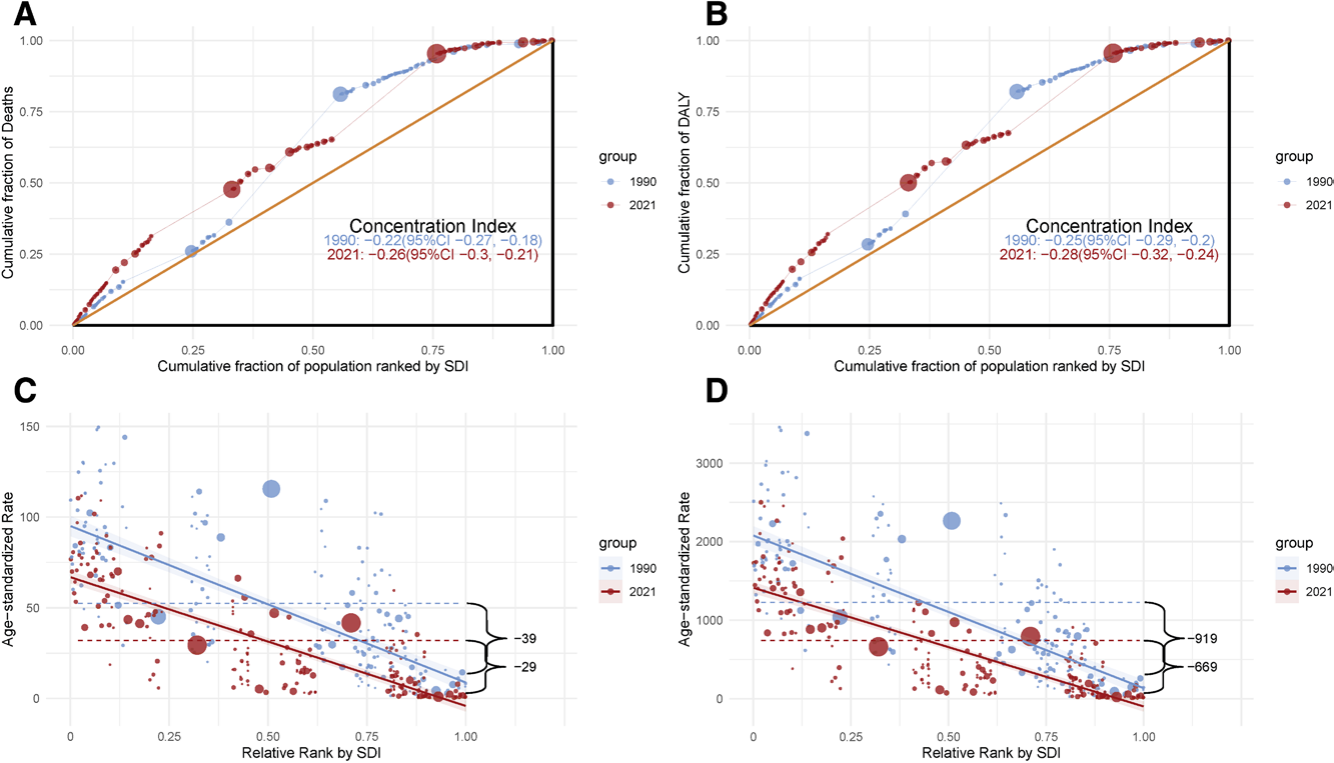
Concentration index and Slope Index of ASMR and DALYs attributable to air pollution across SDI levels in 2021. (A) Concentration index analysis of ASMR. (B) Concentration index analysis of ASDR. (C) Slope index of ASMR. (D) Slope index of ASDR. ASDR = age-standardized disability-adjusted life-year rate, ASMR = age-standardized mortality rate, DALYs = disability-adjusted life-years, SDI = sociodemographic index.

This study systematically analyzed the trends in air pollution-related stroke burden across regions with different SDI levels from 1990 to 2021. In 2021, the middle SDI region exhibited the highest absolute disease burden as shown in Figure 4, with 767,479 deaths (95% UI: 568,987–1,008,333), representing a 17.5% increase compared to 1990. The DALYs in this region reached 16,823,974 person-years (95% UI: 12,428,844–21,838,219), marking a 4.2% increase from 1990. This significant rise is primarily attributed to population growth and aging, as well as the environmental and health challenges posed by rapid urbanization in middle SDI regions. Regarding ASMR, the middle SDI region had the highest ASMR in 1990 (77.73 per 100,000, 95% UI: 63.29–94.43), while the high SDI region had the lowest (12.68 per 100,000, 95% UI: 8.68–17.67). By 2021, the low SDI region emerged with the highest ASMR (53.87 per 100,000, 95% UI: 43.37–63.85), whereas the high SDI region maintained the lowest (3.46 per 100,000, 95% UI: 2.59–4.51). Similarly, for age-standardized DALYs, the low SDI region recorded the highest DALYs in 1990 (1680.76 per 100,000, 95% UI: 1375.99–1984.48), while the high SDI region had the lowest (254.27 per 100,000, 95% UI: 178.36–350.52). In 2021, the low SDI region remained the highest (1162.18 per 100,000, 95% UI: 934.76–1380.38), and the high SDI region continued to have the lowest (80.32 per 100,000, 95% UI: 61.68–102.39). Figure 4 illustrates that all regions exhibited a significant downward trend in ASMR and DALYs (AAPC < 0), indicating global progress in air pollution control and stroke prevention. Notably, the high SDI region demonstrated the most pronounced decline, with an AAPC of −4.14 (95% CI: −4.21 to −4.09) for ASMR and −3.68 (95% CI: −3.74 to −3.64) for DALYs as shown in Figure 4. The persistently low disease burden in high SDI regions can be attributed to their robust public health systems, high healthcare accessibility, and effective air pollution control measures. The concentration index analysis presented in Figure 5 reveals significant absolute disparities in ASMR and ASDR between high and low SDI regions. Specifically, from 1990 to 2021, stroke mortality and ASDR exhibited higher concentration in low SDI regions, indicating a more pronounced health burden in these areas. This trend further exacerbates the inequities in health outcomes across regions with varying levels of development. Slope index further demonstrates that both ASMR and ASDR peaked in low SDI regions, with a significant downward trend observed as SDI values increased. These findings robustly support the notion that advancements in socio-economic development play a pivotal role in reducing mortality rates and mitigating health losses.

### 3.4. National level

**Figure 6.**
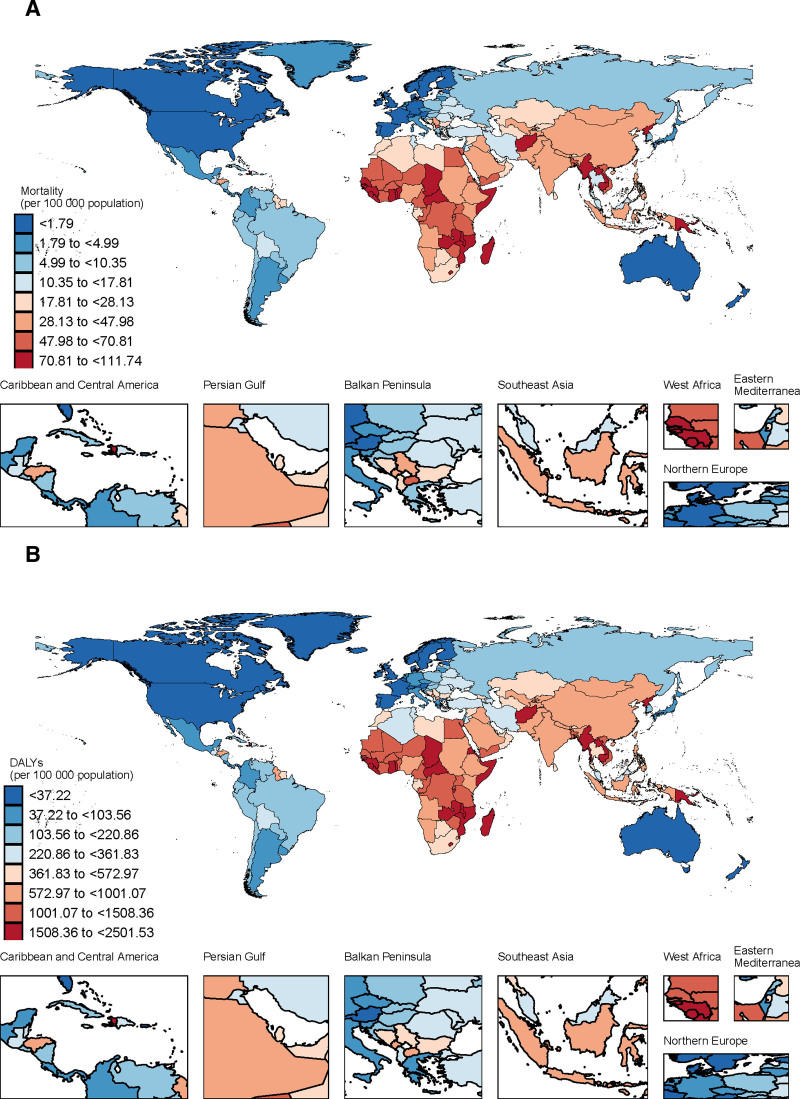
The global disease burden of stroke attributable to air pollution in 204 countries and territories in 2021. (A) ASMR attributable to air pollution. (B) ASDR attributable to air pollution. ASDR = age-standardized disability-adjusted life-year rate, ASMR = age-standardized mortality rate.

In 1990, the global ASMR attributable to air pollution was 48.86 per 100,000 population (95% UI: 39.69–58.76). As shown in File S1, Supplemental Digital Content, https://links.lww.com/MD/Q927, Rwanda (149.56 per 100,000, 95% UI: 114.81–188.66), Laos (149.56 per 100,000, 95% UI: 114.81–188.66), and Myanmar (144.02 per 100,000, 95% UI: 111.99–179.73) exhibited the highest ASMRs globally. The global age-standardized DALYs were 1073.52 per 100,000 (95% UI: 877.41–1276.32), with Laos (3457.62 per 100,000, 95% UI: 2647.1–392.37), Rwanda (3417.13 per 100,000, 95% UI: 2610.19–4383.95), and Myanmar (3457.62 per 100,000, 95% UI: 2647.1–392.37) reporting the highest DALYs. These countries are predominantly located in Southeast Asia and sub-Saharan Africa. By 2021, the global ASMR had declined to 23.74 per 100,000 (95% UI: 18.26–29.8). The national distribution of ASMRs is illustrated in Figure 6, with Guinea-Bissau (111.74 per 100,000, 95% UI: 82.13–139.79), Mozambique (110.42 per 100,000, 95% UI: 80.64–142.49), and the Solomon Islands (111.74 per 100,000, 95% UI: 83.62–139.67) recording the highest ASMRs. The global age-standardized disability-adjusted life years (DALYs) decreased to 523.3 per 100,000 (95% UI: 407.96–645.58). The national distribution of age-standardized DALYs is depicted in Figure 6, with Mozambique (2501.53 per 100,000, 95% UI: 1791.02–3209.44), the Solomon Islands (2454.62 per 100,000, 95% UI: 1854.97–3154.25), and Guinea-Bissau (3377.7 per 100,000, 95% UI: 2606.82–4265.79) exhibiting the highest DALYs. These countries are located in West sub-Saharan Africa, East sub-Saharan Africa, and Oceania, respectively, consistent with regional analysis results, indicating these areas had the highest air pollution-related stroke mortality and DALYs globally in 2021. This phenomenon may be associated with genetic susceptibility, increasing prevalence of hypertension and diabetes, limited healthcare resources, and insufficient awareness of stroke risk factors. From 1990 to 2021, global ASMR and DALYs showed significant declines, particularly in high-income and upper-middle-income countries, demonstrating the effectiveness of public health interventions and improvements in healthcare resources. The most substantial ASMR reductions were observed in Estonia (−5.82, 95% CI: −5.92 to −5.74), Luxembourg (−8.02, 95% CI: −8.16 to −7.91), and Maldives (−7.94, 95% CI: −8.09 to −7.82). The most significant DALYs reductions were recorded in Estonia (−9.27, 95% CI: −9.6 to −8.99), Maldives (−8.48, 95% CI: −8.6 to −8.39), and Luxembourg (−7.9, 95% CI: −8.03 to −7.8). Estonia and Luxembourg, classified as high-income North American and European regions, possess well-developed healthcare systems and robust public health infrastructure. Although located in South Asia, Maldives, with its thriving tourism industry and relatively high economic development, is categorized as an high-middle SDI region. These data reflect the complexity and diversity of air pollution-related stroke epidemiology across different nations.

### 3.5. Burden of ambient air pollution based on sex

**Figure 7.**
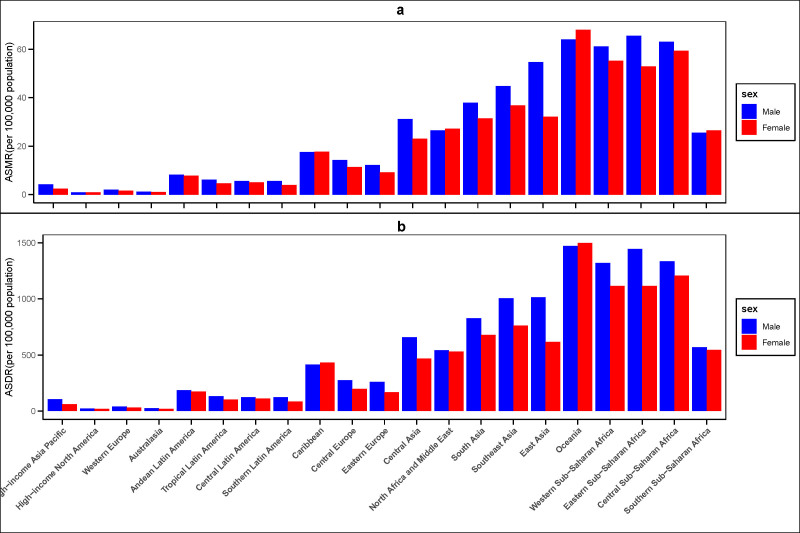
The global disease burden of stroke attributable to air pollution across 21 GBD regions ranked by SDI for both sexes in 2021. (A) ASMR. (B) ASDR. ASDR = age-standardized disability-adjusted life-year rate, ASMR = age-standardized mortality rate, GBD = the Global Burden of Disease, SDI = sociodemographic index.

This study, based on data from 2021, reveals significant gender disparities in the health impacts of air pollution on stroke patients. The results indicate that the ASMR among male patients was 28.31 per 100,000 (95% UI: 21.42–35.65), significantly higher than that of female patients, which was 20.06 per 100,000 (95% UI: 15.62–25.22). The DALYs for male patients were 619.37 per 100,000 (95% UI: 471.91–777.85), compared to 439.00 per 100,000 (95% UI: 345.08–549.03) for female patients. These findings suggest that males generally bear a higher burden of stroke-related health impacts attributable to air pollution compared to females. However, geographic distribution analysis revealed a notable exception. As illustrated in Figure 7, the Oceania region exhibited a reverse trend. In this region, the ASMR for female stroke patients was 68.1 per 100,000 (95% UI: 50.24–89.14), exceeding that of male patients, which was 64.11 per 100,000 (95% UI: 45.03–85.57). Similarly, the DALYs for female patients were 1498.49 per 100,000 (95% UI: 1109.69–1999.05), higher than the 1471.12 per 100,000 (95% UI: 1029.39–1940.88) observed in male patients.

## 4. Discussion

Stroke, as an acute cerebrovascular event, is primarily characterized by the sudden rupture or obstruction of cerebral blood vessels, leading to the interruption of blood supply to the brain and subsequent brain tissue damage. Pathologically, stroke can be classified into 3 main types: cerebral infarction, cerebral hemorrhage, and subarachnoid hemorrhage.^[8]^ According to the 2021 GBD study, stroke remains one of the leading causes of mortality and disability worldwide. Specifically, ischemic stroke accounts for approximately 69,944,885 deaths annually (95% UI: 64,788,695–75,009,603), making it the second leading cause of death globally. Furthermore, stroke contributes to a substantial burden of DALYs, with an estimated 44,962,167 DALYs (95% UI: 35,020,339–55,467,023), further emphasizing its profound impact on global health.^[1]^ In 2019, the global economic loss attributed to stroke reached $2059.67 billion, accounting for 1.66% of the global GDP, highlighting the profound economic implications of this condition beyond individual health.^[9]^ The GBD database also indicates that air pollution is a critical factor contributing to the burden of stroke. Globally, over 90% of the population resides in areas where air quality exceeds the World Health Organization’s annual average PM2.5 standard of 10 μg/m³.^[10]^ In 2017, approximately 2.94 million deaths worldwide were attributed to environmental PM2.5 air pollution, with the burden of disease being particularly severe in low and middle-income countries such as China and India.^[4]^ Stroke not only imposes a substantial health burden globally but also results in significant economic losses. Air pollution, as a major risk factor, exacerbates this issue, especially in low and middle-income countries. Therefore, preventive and control strategies for stroke, particularly measures to reduce air pollution, are crucial for alleviating the global burden of disease.

This study systematically analyzed the association between air pollution and the burden of stroke from 1990 to 2021 based on data from the GBD study. The results revealed a significant upward trend in the absolute burden of stroke-related deaths and DALYs attributable to air pollution on a global scale. This phenomenon is primarily attributed to structural factors such as population growth and aging, indicating that the absolute scale of stroke burden caused by air pollution continues to expand worldwide. However, it is noteworthy that the ASMR and ASDR for stroke both exhibited a significant downward trend during the same period. This finding aligns with previous studies on the global burden of ischemic stroke from 1990 to 2021, which reported an increase in the absolute burden of ischemic stroke in 2021 but a reduction in its relative burden. Specifically, the age-standardized prevalence rate, age-standardized incidence rate, ASMR, and ASDR for ischemic stroke all declined between 1990 and 2021.^[11–14]^ These changes reflect advancements in medical technology, strengthened public health interventions, and the positive effects of local air pollution control policies. Although the absolute burden of stroke continues to rise, the improvement in age-standardized metrics demonstrates significant progress in global stroke prevention and control efforts.

At the regional level, the burden of stroke attributable to air pollution exhibited significant disparities. In 2021, East Asia, South Asia, and Southeast Asia recorded the highest numbers of deaths and DALYs. These regions have undergone rapid industrialization and urbanization in recent decades, particularly in countries such as China, India, and Southeast Asian nations. Their heavy reliance on fossil fuels, including coal and petroleum, coupled with relatively low adoption rates of clean energy, has resulted in persistently high levels of pollutant emissions. Industrial emissions, vehicular exhaust, and construction dust are the primary sources of air pollution in these areas. Additionally, historically high population densities in these regions have exacerbated both pollutant emissions and exposure risks. In contrast, Oceania, Sub-Saharan Central Africa, and Sub-Saharan East Africa exhibited the highest ASMR and ASDR, primarily due to economic underdevelopment, limited healthcare resources, and harsh climatic conditions. At the SDI level, middle SDI regions (e.g., China, India) are currently at the peak of industrialization, facing dual pressures from aging populations and industrial pollution. In 2021, these regions demonstrated the highest absolute disease burden, with deaths and DALYs increasing by 17.5% and 4.2%, respectively, compared to 1990. High SDI regions consistently maintained the lowest disease burden, largely attributable to their robust public health systems and effective air pollution control measures. The fitted curves between ASMR, ASDR, and SDI revealed a significant downward trend with increasing SDI values, further confirming the positive role of socioeconomic development in reducing disease burden. At the national level, countries with the highest ASMR and DALYs in 2021 were predominantly located in Sub-Saharan West Africa, Sub-Saharan East Africa, and Oceania, including Guinea-Bissau, Mozambique, and the Solomon Islands. In contrast, high-income countries such as Estonia, Luxembourg, and the Maldives showed the most pronounced declines in ASMR and DALYs, reflecting the advantages of their healthcare resources and public health systems. High-income countries have effectively mitigated the impacts of air pollution on stroke through policy optimization, economic transformation, and cultural advancements, while low-income countries require enhanced pollution control, improved healthcare resource allocation, and increased health literacy. Gender analysis revealed that males generally bore a higher burden of stroke attributable to air pollution compared to females, with the exception of Oceania, where females exhibited a higher burden. This anomaly may be attributed to economic inequalities in parts of Oceania, where low-income groups, particularly women, are more likely to be exposed to outdoor air pollution sources (e.g., residing near industrial zones). Additionally, women in this region may be more likely to engage in service-oriented occupations such as education and nursing, which often involve prolonged indoor activities with insufficient health protection measures, thereby increasing exposure to indoor air pollution. Furthermore, women may undertake more household responsibilities, such as cooking and cleaning, further elevating their exposure to indoor air pollutants. These findings underscore the importance of considering gender differences and their underlying socioeconomic and occupational exposure factors when formulating air pollution control and stroke intervention strategies.

Air pollution, recognized as one of the most critical environmental risk factors worldwide, has a significant impact on all-cause mortality.^[15]^ Among various air pollutants, fine particulate matter (PM2.5, with aerodynamic diameters <2.5 µm) poses a major public health challenge both in China and globally.^[15,16]^ A substantial body of epidemiological research has recently focused on exploring the potential links between air pollution and stroke, yielding a series of important findings. A prospective cohort study involving 117,575 healthy individuals demonstrated a significant positive correlation between PM2.5 exposure and stroke incidence. Specifically, each 10 μg/m³ increase in PM2.5 concentration was associated with a 13% increase in overall stroke risk (HR: 1.13, 95% UI: 1.09–1.17), with ischemic and hemorrhagic stroke risks increasing by 20% (HR: 1.20, 95% UI: 1.15–1.25) and 12% (HR: 1.12, 95% UI: 1.05–1.20), respectively. The study also identified a near-linear exposure–response relationship between PM2.5 exposure and stroke incidence over a broad concentration range of 31.2–97.0 μg/m³.^[17]^ These findings are consistent with meta-analyses of cohort studies conducted in North America and Europe (HR: 1.46, 95% UI: 1.021–1.109), further validating the significant impact of PM2.5 exposure on stroke risk.^[18]^ Despite the substantial evidence supporting the positive correlation between PM2.5 exposure and stroke, certain inconsistencies remain across studies. For instance, a smaller-scale short-term follow-up cohort study in China found a statistically significant association only with ischemic stroke under low PM2.5 exposure conditions (mean 35.8 ± 2.4 μg/m³), with no significant link observed with hemorrhagic stroke.^[19]^ These discrepancies may be attributed to variations in sample size, follow-up duration, PM2.5 exposure ranges, and population characteristics. Additionally, some case-crossover studies have failed to observe significant associations between PM2.5/O_3_ and stroke recurrence risk, potentially due to differences in ventilation conditions or air conditioning prevalence.^[20]^ To consolidate the diverse findings, a 2021 meta-analysis involving 23 million participants across 68 studies provided more comprehensive evidence in this field.^[21–24]^ The study confirmed that exposure to air pollutants, including PM2.5, SO2, and NO2, was positively correlated with stroke incidence, hospitalization risks (involving PM2.5, PM10, SO2, NO2, CO, and O3), and mortality rates (involving PM2.5, PM10, SO2, and NO2). Furthermore, a 2023 meta-analysis published in Neurology, which included 18,035,408 ischemic stroke patients from 110 studies, indicated that short-term exposure to air pollution might be associated with an increased stroke risk, with PM2.5 exposure showing significant correlations with both stroke incidence (RR: 1.15, 95% UI: 1.13–1.17) and mortality (RR: 1.09, 95% UI: 1.04–1.15).^[25]^ These findings contribute critical scientific evidence for developing precise prevention and treatment strategies for stroke related to air pollution. However, the heterogeneity in study results underscores the need for future research to explore differences across regions, populations, and pollutant types to optimize intervention measures to reduce the global stroke burden. Additionally, there is a pressing need for long-term studies on the effects of low-concentration PM2.5 exposure and the interaction of air pollution with other environmental factors to provide more comprehensive evidence for public health policy formulation.

Numerous large-scale studies have robustly demonstrated a significant positive correlation between chronic exposure to air pollution and the incidence of coronary and cerebrovascular events.^[26,27]^ These studies indicate that even in high-income countries where air pollution levels conform to international standards, a slight increase in air pollutants is closely associated with a higher risk of cerebrovascular diseases, including both hemorrhagic and ischemic strokes.^[26]^ While the pathophysiological mechanisms by which acute exposure to air pollution triggers acute cardiovascular events have been extensively investigated, its mechanisms related to stroke remain incompletely understood. PM2.5, upon entering the respiratory tract, accumulates in the terminal lung tissues, where it induces pulmonary oxidative stress and inflammatory responses. The ensuing inflammatory mediators can traverse the air–blood barrier, leading to systemic inflammation and cerebral tissue damage. Typically, short-term exposure to air pollutants may serve as a trigger for stroke. Research has demonstrated that the exposure can ``increase’’ heart rate variability, alter vagal tone, exacerbate atrial ischemia, and induce pressure changes, thereby precipitating atrial fibrillation and subsequently leading to cardiogenic ischemic stroke.^[26–29]^ Additionally, studies have confirmed that short-term increases in air pollutants elevate the incidence of premature ventricular contractions in individuals with existing cardiovascular diseases, supporting the link between short-term air pollution exposure and cardiogenic ischemic stroke through arrhythmias.^[30]^ Furthermore, air pollution induces endothelial dysfunction, contributing to ischemic stroke through the formation of cardiogenic emboli rather than direct effects on cerebral vasculature.^[31]^ PM exposure has been associated with blood pressure fluctuations, vascular function, atherosclerotic plaque stability, carotid artery thickness, and cerebral vascular resistance, all of which may increase the risk of atherosclerotic-related strokes. Sudden increases in blood pressure or endothelial activation can also precipitate lacunar or hemorrhagic strokes.^[32–34]^ In a murine model of atherosclerosis, PM2.5 exposure led to vascular dysfunction, increased the number of immune cells within plaques, and activated reactive oxygen species formation pathways, thereby enhancing plaque vulnerability.^[35,36]^ Similarly, exposure to diesel exhaust altered vascular tone and promoted inflammatory responses and the production of inflammatory mediators within plaques. Clinically, a meta-analysis further substantiated the impact of PM2.5 on the pathological progression of atherosclerosis, showing a positive correlation between PM concentration and carotid intima-media thickness (IMT). Specifically, for every 10 μg/m^3^ increase in PM concentration, IMT increased by 16.8 μm. Notably, reductions in PM2.5 levels were associated with a gradual decrease in IMT, suggesting a pathologically causal relationship between PM2.5 and carotid atherosclerosis formation.^[36]^ In summary, short-term exposure to air pollution, particularly PM2.5, increases the risk of cerebrovascular events through multiple mechanisms, including cardiogenic stroke, atherosclerotic-related stroke, and hemorrhagic stroke. Long-term exposure (ranging from months to years) to air pollutants poses significantly greater health risks compared to short-term exposure, involving a multitude of complex pathophysiological processes. Prolonged exposure to air pollutants can persistently activate the immune system, leading to a chronic low-grade inflammatory state. The continuous release of inflammatory cytokines (such as IL-6, TNF-α, and CRP) damages vascular endothelial cells, promoting the formation and progression of atherosclerosis, thereby increasing the risk of stroke.^[35,36]^ Free radicals and reactive oxygen species in air pollutants induce sustained oxidative stress, resulting in lipid peroxidation of cell membranes, protein denaturation, and DNA damage. Persistent oxidative stress exacerbates vascular endothelial dysfunction, facilitating thrombus formation and atherosclerosis.^[37,38]^ Endothelial dysfunction is a critical pathological basis for atherosclerosis and stroke. Chronic exposure to air pollution persistently impairs vascular endothelial cells, reducing the bioavailability of nitric oxide and impairing vasodilation.^[39]^ Long-term exposure to air pollution, particularly fine PM2.5, is closely associated with the development of chronic hypertension. Hypertension, a major risk factor for stroke, leads to vascular wall thickening, reduced elasticity, and an increased risk of stroke over time.^[35,37]^ Continuous exposure to air pollution promotes platelet activation and the release of coagulation factors, maintaining a chronic hypercoagulable state, which predisposes to thrombus formation, blocking cerebral blood vessels, and causing ischemic stroke.^[36,40]^ Furthermore, chronic exposure to air pollution disrupts the balance of the autonomic nervous system, leading to sustained sympathetic activation and parasympathetic inhibition. This imbalance may cause persistent blood pressure fluctuations and arrhythmias, thereby elevating stroke risk.^[39]^ Air pollutants accelerate the progression of atherosclerosis by inducing inflammation, oxidative stress, and endothelial dysfunction, promoting lipid deposition and plaque formation, and increasing the risk of cerebrovascular events.^[35,37]^ Certain air pollutants, such as heavy metals and polycyclic aromatic hydrocarbons, can cross the blood–brain barrier, exerting direct toxic effects on the central nervous system, leading to neuronal damage and cerebral dysfunction, thereby increasing stroke risk.^[36,40]^ In summary, long-term exposure to air pollutants increases the risk of stroke through multiple interconnected mechanisms, including inflammatory responses, oxidative stress, endothelial dysfunction, hypertension, hypercoagulability, autonomic nervous system imbalance, atherosclerosis, and central nervous system toxicity. These mechanisms collectively impact the cerebrovascular system, ultimately contributing to the occurrence of stroke.

The analysis conducted in this study, based on the GBD database, offers a comprehensive assessment of global disease burden; however, several limitations must be acknowledged to ensure accurate interpretation of the results. Firstly, the GBD database primarily relies on statistical data reported by individual countries, which introduces variability due to significant differences in data collection capabilities, reporting standards, and quality across regions. Secondly, the GBD analysis is predominantly based on historical data, which limits its ability to predict the future impacts of policies or interventions (e.g., air pollution control measures, public health initiatives) on disease burden. Additionally, the analysis depends heavily on statistical models and assumptions, such as linear exposure–response relationships, which may not fully align with real-world conditions. For instance, air pollution exposure is often estimated using broad-scale monitoring data or modeling, which may not accurately reflect individual exposure levels, particularly in the context of indoor air pollution or occupational exposures. The reasons for the absence of individual data are as follows: restrictions on data collection protocols: GBD integrates macro data sources such as annual health statistics reports and disease registration systems of various countries; privacy protection requirements: strictly comply with international privacy regulations such as the General Data Protection Regulation; methodological framework design: spatial-time smoothing processing is carried out based on the Bayesian hierarchical model. Finally, the multifactorial nature of disease attribution presents a significant challenge. Stroke is influenced by a combination of genetic, lifestyle, and underlying health factors, with air pollution being one of many contributing elements. The GBD analysis may struggle to fully disentangle the effects of these confounding factors, potentially leading to biased estimates of the role of air pollution in stroke burden.

In summary, air pollution remains a significant environmental factor contributing to the global burden of stroke. Although age-standardized rates associated with air pollution have shown a declining trend, the absolute burden of stroke remains substantial, with notable disparities across regions, countries, SDI levels, and genders. Moving forward, stroke prevention and control strategies should not only target traditional risk factors such as hypertension, diabetes, and unhealthy lifestyles but also emphasize the critical role of air pollution. Specifically, high-risk populations should reduce exposure to polluted environments and adopt effective protective measures. Additionally, tailored prevention and treatment strategies should be developed to address the specific needs of different countries, regions, and populations, particularly in low and middle-income countries, to further reduce the global burden of stroke and promote health equity.

## Acknowledgments

We extend our sincere gratitude to the contributors of the Global Burden of Disease, Injury, and Risk Factor Study 2021 for their invaluable efforts.

## Author contributions

**Conceptualization:** Changqiang Feng.

**Data curation:** Songxin Zhong, Changqiang Feng.

**Formal analysis:** Changqiang Feng.

**Funding acquisition:** Chao Xiao, Yanni Lin, Chao Qin.

**Investigation:** Chao Xiao, Rida Li, Yanni Lin.

**Methodology:** Chao Xiao, Rida Li, Xiaomin Feng.

**Project administration:** Rida Li, Xiaomin Feng.

**Resources:** Xiaomin Feng, Shizhan Li.

**Software:** Shizhan Li, Zukun He, Yijiu Lu.

**Supervision:** Shizhan Li, Zukun He, Yijiu Lu.

**Validation:** Zukun He, Yijiu Lu, Jianqing Zhu.

**Visualization:** Songxin Zhong, Jianqing Zhu.

**Writing – original draft:** Songxin Zhong, Yanni Lin.

**Writing – review & editing:** Songxin Zhong, Chao Qin.

Supplemental digital content “Table S1 and S2” are available for this article (https://links.lww.com/MD/Q927).

## Supplementary Material


