# Supplementary material for: Global burden of stroke attributable to air pollution (1990–2021): An analysis of GBD 2021 data
Source: Medicine (Baltimore). 2025 Dec 19;104(51):e46419. doi: 10.1097/MD.0000000000046419 (PMC12727343; doi:10.1097/MD.0000000000046419)
Supplement: Supplementary file 1 [file medi-104-e46419-s001.pdf]

Methods:

1. **AAPC**

To assess the trends in age-standardized rates (ASR) of ischemic stroke incidence, DALYs,mortality, and prevalence, the study utilized the annual percent change(APC) and average annual percent change (AAPC) . The ASR was computed per 100,000 individuals utilizing the subsequent formula:ASR $= \frac{\sum_{i=1}^{A} a_i w_i}{\sum_{i=1}^{A} w_i} \times 100{,}000$, where ai and wi denote age-specific rates and the number of persons (or weight) in the same age subgroup of the chosen reference standard population (where i denotes the ith age class), respectively.

2. **Jointpoint regression**

In this study, Jointpoint regression was employed to analyze the temporal trends of disease burden, aiming to identify significant change points in indicators such as incidence and mortality. Initially, relevant data from the Global Burden of Disease (GBD) study were included and subjected to quality control to ensure data integrity and accuracy. Subsequently, the Jointpoint software (Version 5.3) was utilized for analysis. A combination of grid search and likelihood ratio tests was applied to automatically determine the optimal number and location of jointpoints. Model fit was evaluated using the Akaike Information Criterion (AIC) and Bayesian Information Criterion (BIC) to select the most appropriate model. Finally, the Annual Percentage Change (APC) and its 95% Confidence Interval (CI) were calculated for each time period to quantify the magnitude of trend changes, and the statistical significance of jointpoints was assessed via likelihood ratio tests ($P<0.05$ was considered statistically significant). Specific calculation methods can be seen in this study by Kim. [1]

3. **Decomposition analysis of deaths and DALYs**

We conducted a decomposition analysis of changes in DALYs from 2010 to 2019, decomposing changes in all-age cause-specific DALYs attributable to all risk factors and individual risk factors due to changes in population growth, population age structure, exposure to the given risk for a disease, and risk-deleted death and DALY rates. In this case, risk-deleted rates are the rates obtained after removing the effect of a risk factor or combination of risk factors — in other words, observed DALY rates multiplied by one minus the PAF for the risk or set of risks. Our decomposition analyses draw from methods developed by Das Gupta to provide a computationally tractable solution for isolating drivers of burden changes whereby all combinations of possible pathways are averaged across factors.[2]

Attributable burden was determined, following the methods of Das Gupta, as a product of three factors such that: where Tasgt represents the attributable burden at year ; Asgt is the age-specific population size for a given age group a, sex s, and location g at year t; Basgt is the underlying rate of the outcome unrelated to the risk factor or observed rate, multiplied by 1− PAFfor a given age group a, sex s, and location g at year t; and Casgt is the ratio of the attributable burden to the underlying rate, which reflects the risk exposure effect for a given age group a, sex s, and location g at year ; defined as PAF/ (1-PAF)when decomposing attributable burden to a risk. Risk exposure effects for individual risk factors are scaled such that they sum to the all-risk exposure effect by location, age, sex, and cause accounting for mediation. This process allows for aggregation of risks; the exposure for all risks for a disease can be split into exposure to metabolic, behavioural, and environmental risks. The contribution of each factor to total change in attributable burden was determined by changing the level of one factor from time t0 to t1 – here 2010 to 2019 – with all other factors held constant. Thus, the effect of any of the three factors, for example Aasgt on the change of the attributable burden between 2010 (A10) and 2019 (A17) is calculated as:

where EA is the proportion of change due to factor 6, and the subscripts for each factor in the equation denote the year for each estimate. Because the effect depends on the order of entry of the factor, we calculated the average of all combinations of the three factors. The proportion of change due to factor Asgt, the age-specific population size for a given age group a, sex s, and location g at year t, is then further split, setting change in population growth equal to the percentage change in the all-age population from time t0 to t1 and change in population age structure to the residual, giving four factors. The calculation formula is as follows□

$$E_A = (A_{19} - A_{10})\left(\frac{B_{10}C_{10} + B_{19}C_{19}}{3} + \frac{B_{10}C_{19} + B_{19}C_{10}}{6}\right)$$

4. **health inequality analysis**

In health inequality analysis, the Slope Index of Inequality (SII) and Concentration Index (CI) are employed to assess disparities in disease burden across socioeconomic statuses.

- **Slope Index of Inequality (SII):** Measures the absolute difference in health outcomes between socioeconomic groups. It quantifies the health gradient from the lowest to the highest socioeconomic strata (e.g., income, education, or Socio-demographic Index, SDI) using a regression model, reflecting the absolute magnitude of health inequality. The formula is:
  uj=β0+β1; Rj, for j=1 to J
- uj: The average health level (e.g., incidence rate, mortality rate) of the j-th socioeconomic group.
- Rj: The relative rank of this group in the population (cumulative proportion, ranging from 0 to 1).
- β1 (i.e., SII): The absolute difference in health levels between the highest and lowest socioeconomic groups.
- **Concentration Index (CI):** Evaluates the relative association between health indicators and socioeconomic status, based on the Lorenz curve principle. It measures the degree of inequality in health distribution across a population, with a range of [−1,1]:

CI = 0: Perfectly equitable health distribution.

CI < 0: Health outcomes are concentrated among lower socioeconomic groups (e.g., higher disease burden in impoverished populations).

CI > 0: Health outcomes are concentrated among higher socioeconomic groups (e.g., better access to healthcare for wealthier individuals).

$$C = \frac{2}{u} cov(h_i, r_i)$$

hi: individual health status.

Ri: Individual socioeconomic ranking (rank order after sorting by income or SDI)

u: Population-average health level

[1] Kim HJ, Fay MP, Feuer EJ, Midthune DN. Permutation tests for joinpoint regression with applications to cancer rates. Stat Med 2000;19:335-51 (correction: 2001;20:655).

[2] Das gupta p. Standardization and decomposition of rates: A user’s manual. Washington d.C.: U.S. Bureau of the census, 1993.

Table 1: The mortality cases and ASMR of stroke attributable to air pollution in 1990 and 2021 and its trends.

	Mortality				
	1990 counts (95%UI)	2021 counts (95%UI)	1990 ASR (95%UI)	2021ASR (95%UI)	AAPC (95%CI)
Global	1755017.25 (1434138.6-2094574.28)	1989686.32 (1530479.07-2493237.87)	48.86 (39.69-58.76)	23.74 (18.26-29.8)	-2.43 (-2.49, -2.37)
Sex					
Male	865991.61 (696794.86-1043249.92)	1055764.05 (798130.97-1328422.25)	54.98 (44.18-66.38)	28.31 (21.42-35.65)	-2.17 (-2.27, -2.12)
Female	889025.64 (713428.28-1079934.85)	933922.27 (726991.02-1174376.35)	44.05 (35.34-53.63)	20.06 (15.62-25.22)	-2.64 (-2.69, -2.58)
SDI quintiles					
High SDI	141013.07 (96959.75-196349.16)	81577.09 (60005.23-107352.29)	12.68 (8.68-17.67)	3.46 (2.59-4.51)	-4.14 (-4.21, -4.09)
High-middle SDI	487241.13 (377423.01-608683.31)	388996.79 (292154.97-512267.73)	55.94 (42.8-70.51)	20.01 (15.04-26.35)	-3.49 (-3.57, -3.38)
Middle SDI	653037.38 (533201.82-791568.52)	767478.72 (568986.82-1008332.62)	77.73 (63.29-94.43)	32.11 (23.83-42.15)	-2.96 (-3.06, -2.88)
Low-middle SDI	330493.89 (269286.1-389627.09)	525073.74 (411295.13-629590.89)	63.85 (52.03-75.39)	41.42 (32.35-49.81)	-1.4 (-1.47, -1.33)
Low SDI	141329.84 (114505.34-167203.68)	225105.31 (180623.01-266855.14)	75.55 (61.78-89.16)	53.87 (43.37-63.85)	-1.08 (-1.11, -1.04)
21 GBD Region					
Andean Latin America	5988.31 (4641.96-7536.62)	4574.81 (2940.26-6594.78)	31.41 (24.43-39.47)	7.98 (5.14-11.53)	-4.3 (-4.43, -4.17)
Australasia	540.02 (18.79-1585.98)	734.01 (421.74-1118.67)	2.45 (0.09-7.21)	1.16 (0.67-1.76)	-2.18 (-2.68, -1.57)
Caribbean	7336 (5266.22-9948.87)	9525.2 (6722.17-12844.07)	29.83 (21.26-40.66)	17.65 (12.46-23.74)	-1.63 (-1.69, -1.58)

Central Asia	17255.68 (10011.94-24923.25)	17967.56 (13389.17-23647.28)	40.79 (23.72-58.7)	26.41 (19.58-34.67)	-1.42 (-1.56, -1.26)
Central Europe	69046.29 (40866.74-97056.02)	30559.38 (22440.48-43933.07)	52.05 (30.69-72.97)	12.75 (9.36-18.31)	-4.5 (-4.58, -4.43)
Central Latin America	13359.35 (8833.6-18265.5)	12760.02 (8665.92-18238.96)	18.69 (12.32-25.57)	5.35 (3.63-7.65)	-3.96 (-4.03, -3.88)
Central Sub-Saharan Africa	13800.97 (10089.66-17571.34)	24815.82 (17681.96-33436.8)	80.35 (60.02-102.13)	61.34 (44.12-83.87)	-0.88 (-0.92, -0.83)
East Asia	745480.91 (590871.9-913565.85)	815599.15 (593057.5-1078387.4)	113.43 (90.37-138.12)	41.52 (30.4-54.8)	-3.24 (-3.37, -3.14)
Eastern Europe	107766.48 (54753.93-167053.49)	37507.96 (23484.03-57055.17)	43.72 (22.04-67.76)	10.51 (6.58-15.99)	-4.57 (-4.86, -4.21)
Eastern Sub-Saharan Africa	54523.12 (44209.9-65905.34)	80842.5 (64052.05-97949.54)	87.5 (71.18-105.44)	58.85 (46.7-71.65)	-1.27 (-1.29, -1.26)
High-income Asia Pacific	21774.94 (6433.08-43053.19)	19868.02 (11481.7-29960.83)	12.01 (3.47-23.89)	3.28 (1.91-4.87)	-4.09 (-4.2, -3.96)
High-income North America	15178.67 (5862.97-27020.51)	6657.15 (3284.92-11054.1)	4.15 (1.6-7.39)	0.93 (0.46-1.54)	-4.74 (-4.91, -4.53)
North Africa and Middle East	66944.81 (51777.35-82555.9)	99799.6 (76010.02-123439.78)	49.79 (38.19-61.74)	26.85 (20.57-33.21)	-2.07 (-2.15, -1.98)
Oceania	2136.1 (1586.45-2756.21)	4039.19 (2928.21-5305.97)	90.59 (68.32-115.86)	66.15 (48.16-86.24)	-1 (-1.05, -0.97)
South Asia	250053.47 (198667.32-297583.77)	449965.85 (359354.37-534837.78)	51.88 (41.08-62.02)	34.5 (27.52-41.24)	-1.34 (-1.5, -1.2)
Southeast Asia	188257.45 (148822.59-226024.13)	230470.01 (160755.72-307502.86)	87.72 (69.02-105.65)	40.69 (28.46-54.51)	-2.5 (-2.6, -2.45)
Southern Latin America	8229.9 (4398.43-12775.34)	4282.39 (2640.25-6544.49)	19.18 (10.26-29.87)	4.72 (2.91-7.22)	-4.44 (-4.6, -4.32)
Southern Sub-Saharan Africa	7477.26 (5481.78-9569.01)	12841.61 (9485.8-16770.14)	31.76 (23.12-40.89)	26.54 (19.46-34.71)	-0.55 (-0.62, -0.47)

Tropical Latin America	23271.94 (13737.15-34917.32)	13206.9 (7714.69-20242.01)	29.95 (17.95-44.6)	5.32 (3.11-8.16)	-5.48 (-5.59, -5.4)
Western Europe	75944.06 (36734.53-125859.32)	22532.83 (15121.14-32075.6)	12.63 (6.09-20.95)	1.86 (1.25-2.65)	-6.05 (-6.17, -5.95)
Western Sub-Saharan Africa	60651.52 (47996.54-72991.88)	91136.37 (70305.42-112049.61)	82.86 (65.72-99.96)	58.08 (45.13-71.36)	-1.13 (-1.16, -1.11)
204 countries and regions					
Afghanistan	7303.41 (5223.95-9481.03)	6762.42 (4848.33-8925.88)	124.81 (89.99-162.79)	85.7 (61.82-112.42)	-1.22 (-1.28, -1.15)
Albania	1354.28 (1023.88-1685.12)	959.67 (561.83-1598.27)	82.84 (62.46-102.96)	23.49 (13.75-39.01)	-4.11 (-4.33, -3.94)
Algeria	2197.37 (1374.56-3135.94)	4835.42 (3099.48-6979.1)	29.32 (18.45-42.11)	19.61 (12.75-28.27)	-1.25 (-1.36, -1.11)
American Samoa	1.02 (0-4.1)	1.97 (0.17-5.04)	5.81 (0-23.39)	4.86 (0.41-12.39)	-0.47 (-0.57, -0.38)
Andorra	2.29 (0.89-4.35)	1.85 (0.96-2.89)	4.89 (1.92-9.32)	1.04 (0.54-1.62)	-4.98 (-5.12, -4.83)
Angola	2553 (1882.84-3375.47)	3445.97 (2158.47-4842)	85.73 (63.78-112.09)	40.49 (25.67-57.33)	-2.4 (-2.48, -2.33)
Antigua and Barbuda	9.8 (2.25-22.41)	9.26 (3.31-17)	17.1 (3.93-39.11)	9.92 (3.58-18.28)	-1.67 (-1.85, -1.47)
Argentina	5242.99 (2448.95-8701.1)	2345.69 (1204.93-3799.3)	17.49 (8.19-29.02)	4.06 (2.09-6.58)	-4.52 (-4.7, -4.38)
Armenia	713.72 (417.51-1082.22)	677.38 (447.91-940.98)	30.94 (18.02-46.89)	15.73 (10.4-21.84)	-2.17 (-2.38, -1.91)
Australia	459.1 (15.48-1320.26)	640.86 (375.52-955.12)	2.51 (0.09-7.24)	1.19 (0.69-1.76)	-2.39 (-2.93, -0.9)
Austria	1883.69 (970.74-3072.14)	360.94 (242.93-507.12)	15.07 (7.78-24.62)	1.58 (1.08-2.21)	-7.18 (-7.33, -7.01)

Azerbaijan	1367.01 (563.44-2198.06)	1344.31 (705.78-2187.65)	31.81 (12.92-51.01)	17.02 (9.05-27.71)	-2.11 (-2.29, -1.95)
Bahamas	17.31 (2.7-39.84)	27.12 (8.57-54.69)	12.22 (1.91-28.11)	7.53 (2.39-15.13)	-1.57 (-1.7, -1.43)
Bahrain	63.13 (48.38-79.13)	129.47 (95.08-164.88)	62.26 (47.92-79.01)	29.69 (21.9-37.19)	-2.4 (-2.59, -2.22)
Bangladesh	42628.78 (34139.64-52337.93)	82967.64 (61948.13-106291.12)	102.31 (82.26-124.91)	70.09 (52.48-88.73)	-1.08 (-1.28, -0.91)
Barbados	59.06 (15.33-127.36)	62.87 (25.49-112.08)	19.35 (5.04-41.74)	12.14 (4.92-21.61)	-1.48 (-1.67, -1.27)
Belarus	3954.81 (2102.66-6057.53)	1798 (1238.16-2500.17)	31.8 (16.91-48.77)	11 (7.56-15.27)	-3.55 (-3.79, -3.23)
Belgium	2147 (1086.59-3582.8)	550.74 (362.73-766.64)	13.6 (6.89-22.64)	1.86 (1.24-2.57)	-6.27 (-6.36, -6.18)
Belize	18.55 (10.82-28.6)	31.71 (14.54-52.02)	20.16 (11.77-31.08)	11.95 (5.45-19.61)	-1.41 (-1.64, -1.2)
Benin	1711.01 (1362.07-2065.17)	3060.89 (2301.89-3856.4)	97.16 (77.19-117.15)	74.01 (56.97-91.81)	-0.88 (-0.91, -0.85)
Bermuda	2.19 (0-5.85)	1.51 (0.28-3.01)	3.9 (0-10.38)	0.99 (0.18-1.95)	-4.32 (-4.44, -4.21)
Bhutan	95.47 (67.16-129.9)	103.31 (69.3-141.41)	49.38 (33.85-67.03)	18.67 (12.62-25.51)	-3.4 (-3.7, -3.18)
Bolivia (Plurinational State of)	1575.84 (1097.96-2193.23)	1272.17 (779.65-1999.68)	55.68 (39.47-76.75)	16 (9.85-24.96)	-4.07 (-4.14, -3.99)
Bosnia and Herzegovina	2446.79 (1946.84-2982.3)	1795.21 (1186.22-2789.17)	75.3 (59.95-91.85)	27.83 (18.38-43.28)	-3.15 (-3.24, -3.07)
Botswana	309.24 (193.27-421.44)	205.1 (110.73-365.84)	73.23 (46.43-100.34)	18.6 (10.01-32.88)	-4.6 (-4.84, -4.45)
Brazil	22490.48 (13202.35-33937.62)	12683.75 (7422.46-19219.66)	29.68 (17.64-44.44)	5.22 (3.05-7.92)	-5.51 (-5.62, -5.43)

Brunei Darussalam	5.41 (0.35-14.24)	6.77 (1.49-13.84)	6.46 (0.43-17)	2.83 (0.62-5.75)	-2.8 (-3.1, -2.54)
Bulgaria	6650.39 (3628.39-9742.33)	3881.32 (2767.01-5900.73)	70.69 (38.57-103.87)	26.57 (18.91-40.52)	-3.22 (-3.4, -3.02)
Burkina Faso	2137.6 (1654.2-2686.88)	4060.44 (3011.27-5221.98)	60.15 (46.66-75.52)	53.34 (40.28-67.9)	-0.39 (-0.44, -0.34)
Burundi	2692.34 (1970.47-3451.66)	2733.32 (2031.28-3574.05)	130.2 (96.41-167.13)	70.61 (52.88-93.08)	-1.98 (-2.01, -1.95)
Cabo Verde	106.34 (81.42-132.8)	138.52 (95.7-186.35)	45.1 (34.5-56.41)	33.42 (23.05-45.12)	-0.82 (-0.97, -0.67)
Cambodia	4380.46 (3491.61-5296.96)	8007.77 (5983.43-10103.06)	118.93 (94.18-145.32)	83.13 (62.98-104.8)	-1.16 (-1.17, -1.14)
Cameroon	2748.54 (2059.37-3534.13)	6751.41 (4812.55-9240.29)	76.01 (57.31-96.89)	67.51 (49.12-91.07)	-0.4 (-0.45, -0.36)
Canada	973.98 (277-1874.56)	394.12 (143.77-730.46)	3.08 (0.88-5.94)	0.47 (0.18-0.87)	-5.84 (-6.04, -5.62)
Central African Republic	1000.84 (701.33-1320.02)	1572.88 (1065.62-2186.68)	113 (79.85-145.98)	96.89 (67.53-130.09)	-0.5 (-0.52, -0.47)
Chad	1958.06 (1491.23-2459.72)	3815.94 (2798.47-5089.98)	78.07 (58.92-98.03)	80.62 (59.52-105.93)	0.09 (0.06, 0.15)
Chile	2458.81 (1687.75-3277.76)	1711.53 (1125.51-2407.82)	27.28 (18.67-36.5)	6.51 (4.29-9.16)	-4.64 (-4.74, -4.55)
China	727747.96 (576056.53-892072.22)	785932.44 (568789.06-1045155.21)	115.57 (91.96-140.92)	41.72 (30.39-55.35)	-3.29 (-3.42, -3.19)
Colombia	3262.25 (2045.62-4500.41)	1894.16 (1119.31-2958.57)	21.06 (13.27-29.08)	3.39 (2.02-5.3)	-5.81 (-6.01, -5.67)
Comoros	144.41 (104.11-185.49)	206.28 (146.18-267.82)	89.41 (66.12-113.1)	50.36 (36.69-65.3)	-1.88 (-1.93, -1.82)
Congo	849.17 (614.4-1105.14)	1075.23 (726.36-1468.65)	100.85 (74.51-129.54)	53.81 (36.31-72.33)	-2.01 (-2.11, -1.94)

Cook Islands	0.75 (0.03-2.28)	0.44 (0-1.21)	7.08 (0.24-21.61)	1.74 (0-4.8)	-4.34 (-4.42, -4.26)
Costa Rica	178.54 (96.67-259.06)	151.22 (94.67-222.41)	10.92 (5.92-15.89)	2.7 (1.69-3.98)	-4.61 (-4.85, -4.42)
Coted'Ivoire	2419.25 (1852.3-3112.05)	5705.66 (4110.15-7768.82)	79.79 (62.44-100.77)	65.57 (48.36-87.51)	-0.62 (-0.68, -0.57)
Croatia	2013.59 (1034.26-3196.14)	778.85 (557.64-1033.29)	38.74 (19.86-61.64)	7.69 (5.51-10.18)	-5.11 (-5.28, -4.87)
Cuba	1268.02 (480.16-2485.21)	1735.84 (820.29-2924.89)	13.11 (4.97-25.7)	8.33 (3.92-14.04)	-1.33 (-1.48, -1.12)
Cyprus	123.24 (53.51-213.47)	82 (56.22-111.1)	25.24 (11.39-44.2)	5.08 (3.43-6.88)	-5.4 (-5.66, -5.1)
Czechia	4958.39 (2591.02-7999.52)	955.22 (693.23-1288.08)	36.93 (19.36-59.5)	4.06 (2.95-5.47)	-6.85 (-7.09, -6.69)
Democratic People's Republic of Korea	14129.89 (10377.63-18303.37)	27905.01 (21467.15-36355.1)	108.93 (81.58-139.94)	91.08 (69.89-119.01)	-0.58 (-0.61, -0.57)
Democratic Republic of the Congo	9048.41 (6393.85-11853.5)	18433.35 (12911.07-25686.44)	77.31 (55.66-100.96)	68.16 (47.36-95.39)	-0.44 (-0.5, -0.38)
Denmark	861.26 (385.92-1486.06)	237.97 (140.24-359.82)	9.79 (4.39-16.91)	1.72 (1.01-2.6)	-5.82 (-6.01, -5.65)
Djibouti	65.33 (44.2-91.28)	182.44 (114.72-271.32)	64.18 (43.53-86.64)	39.05 (24.38-58.59)	-1.7 (-1.79, -1.63)
Dominica	21.71 (12.54-30.98)	11.52 (5.18-19.86)	37.67 (21.6-53.92)	15.02 (6.75-25.79)	-2.95 (-3.01, -2.89)
Dominican Republic	936.51 (575.9-1308.63)	1048.18 (379.37-1887.72)	29.49 (18.01-40.91)	10.75 (3.89-19.38)	-3.18 (-3.29, -3.1)
Ecuador	1257.19 (846.95-1679.91)	867.47 (505.56-1331.88)	25.85 (17.42-34.57)	5.7 (3.34-8.75)	-4.75 (-5.02, -4.47)
Egypt	17506.55 (13100.75-22685.32)	27653.56 (19223.34-36313.55)	96.88 (73.77-125.17)	66.37 (46.92-86.6)	-1.39 (-1.57, -1.24)

El Salvador	735.69 (590.41-903.03)	385.36 (226.21-562.32)	25.53 (20.51-31.24)	5.83 (3.44-8.53)	-4.6 (-4.8, -4.37)
Equatorial Guinea	158.97 (115.59-203.86)	92.48 (44.31-158.5)	100.89 (75.12-128.2)	24.47 (11.8-41.32)	-4.41 (-4.54, -4.3)
Eritrea	1007.13 (737.82-1311.12)	1469.47 (1023.71-1926.36)	113.24 (84.56-143.58)	70.12 (50.4-90.15)	-1.58 (-1.62, -1.55)
Estonia	462.62 (178.85-835.53)	36.6 (11.61-79.18)	23.34 (9.02-42.06)	1.14 (0.36-2.44)	-9.62 (-9.97, -9.31)
Eswatini	171.67 (122.13-226.73)	200.65 (103.1-332.49)	77.59 (55.95-100.59)	46.7 (24.13-75.23)	-1.61 (-1.66, -1.56)
Ethiopia	14091.69 (11083.04-18620.9)	14377.39 (11058.21-17898.22)	84.15 (66.5-107.6)	39.16 (29.97-48.64)	-2.46 (-2.48, -2.43)
Fiji	155.23 (89.83-213.7)	125.1 (38.46-237.49)	53.94 (31.34-73.52)	21.32 (6.66-40.19)	-3.06 (-3.18, -2.98)
Finland	306.09 (47.91-654.65)	70.82 (9.98-162.02)	4.23 (0.66-9.04)	0.45 (0.06-1.02)	-7.45 (-7.92, -7.13)
France	7611.31 (3529.94-13123.82)	2521.49 (1607.21-3689.08)	8.49 (3.92-14.55)	1.28 (0.83-1.87)	-5.87 (-5.98, -5.78)
Gabon	190.57 (101.36-311.05)	195.92 (108.48-310.25)	38.39 (20.46-61.68)	24.37 (13.53-39.07)	-1.42 (-1.56, -1.27)
Gambia	238.37 (180.22-308.05)	699.75 (508.69-909.55)	83.21 (63.14-105.31)	85.37 (62.51-109.94)	0.19 (0.1, 0.29)
Georgia	2345.16 (881.12-3942.86)	1794.59 (1061.04-2761.32)	40.61 (15.3-68.21)	27.64 (16.47-42.41)	-1.33 (-1.6, -1.05)
Germany	19400.83 (9508.43-31886.33)	4167.05 (2738.72-5912.8)	14.41 (7.06-23.66)	1.73 (1.15-2.45)	-6.67 (-6.91, -6.45)
Ghana	5215.72 (3968.61-6485.32)	11216.38 (8085.09-14393.52)	101.91 (78.74-126.24)	84.06 (61.57-107.58)	-0.64 (-0.71, -0.6)
Greece	3969.06 (2015.5-6472.06)	1827.98 (1296.89-2428.53)	27.89 (14.15-45.46)	5.46 (3.89-7.26)	-5.14 (-5.43, -4.92)

Greenland	1.49 (0.01-4.1)	0.92 (0.04-2.44)	6.41 (0.03-17.83)	1.8 (0.07-4.82)	-3.9 (-4.09, -3.68)
Grenada	33.2 (18.36-51.78)	15.07 (6.11-27.65)	42.25 (23.4-65.74)	15.68 (6.3-28.58)	-3.22 (-3.38, -3.06)
Guam	2.56 (0-7.19)	4.68 (2.15-7.44)	4.78 (0-13.33)	2.22 (1.02-3.54)	-2.69 (-3.2, -2.15)
Guatemala	785.37 (622.05-939.52)	1277.37 (766.83-1802.18)	29.29 (23.36-35.03)	13.2 (8-18.6)	-2.58 (-2.81, -2.4)
Guinea	2375.72 (1795.78-2929.3)	3803.9 (2840.31-4978.41)	81.17 (61.99-100.48)	79.05 (59.92-102.64)	-0.08 (-0.1, -0.06)
Guinea-Bissau	438.33 (327.42-566.58)	632.93 (459.05-809.74)	129.51 (98.7-164.78)	111.74 (82.13-139.79)	-0.46 (-0.48, -0.43)
Guyana	240.73 (137.4-378.06)	152.96 (67.46-261.83)	69.8 (40.04-109.85)	27.76 (12.25-47.12)	-2.92 (-3.06, -2.77)
Haiti	3431.34 (2674.77-4332.13)	5205.8 (3620.9-7179.29)	129.66 (101.05-161.81)	89.68 (62.65-122.01)	-1.17 (-1.19, -1.14)
Honduras	758.99 (580.62-925.86)	2130.43 (1454.7-2820.36)	41.67 (31.69-50.9)	39.69 (27.04-52.91)	-0.18 (-0.31, -0.06)
Hungary	5587.12 (2885.5-9100.91)	1294.22 (905.1-2162.77)	40.91 (21-66.65)	6.03 (4.21-10.07)	-6.13 (-6.33, -5.93)
Iceland	6.67 (0.5-15.87)	2.35 (0.45-5.34)	2.16 (0.16-5.13)	0.33 (0.06-0.75)	-6.13 (-6.44, -5.77)
India	177180.76 (138329.54-214905.94)	317494.69 (252258.71-387424.75)	45.1 (35.18-54.79)	29.42 (23.38-35.89)	-1.43 (-1.54, -1.32)
Indonesia	72539.57 (55925.14-90463.45)	87214.38 (56833.67-123947.6)	88.79 (67.44-111.91)	47.13 (30.54-66.58)	-2.11 (-2.26, -2.03)
Iran (Islamic Republic of)	5563.46 (4287.46-6861.58)	9956.11 (7747.17-12607.66)	31.34 (23.88-38.46)	15.18 (11.7-19.27)	-2.38 (-2.49, -2.29)
Iraq	4462.35 (3086.89-6055.82)	8082.56 (5388.86-11189.93)	60.31 (41.81-81.73)	44.95 (30.73-61.76)	-0.82 (-0.91, -0.7)

Ireland	320.61 (127.21-579.02)	74.84 (38.07-120.24)	8.28 (3.29-14.96)	0.87 (0.44-1.39)	-7.02 (-7.2, -6.86)
Israel	501.75 (273.35-800.54)	410.38 (302.35-528.8)	11.11 (6.07-17.77)	2.93 (2.16-3.76)	-4.46 (-4.87, -3.93)
Italy	15028.46 (8251.94-23481.44)	6211.05 (4413.82-8447.51)	17.2 (9.44-26.88)	3.06 (2.18-4.13)	-5.51 (-5.65, -5.35)
Jamaica	685.02 (410.8-935.16)	449.97 (239.66-706.46)	37.05 (22.22-50.54)	13.66 (7.34-21.44)	-2.83 (-3.7, -2.18)
Japan	11707.17 (2251.81-25735.49)	12521.49 (6497.71-19900.04)	7.65 (1.47-16.84)	2.42 (1.29-3.81)	-3.65 (-3.77, -3.5)
Jordan	255.69 (183.54-335.77)	803.34 (564.66-1058.18)	25.95 (18.52-34.22)	15.68 (11.04-20.59)	-2.08 (-2.32, -1.85)
Kazakhstan	3695.28 (1412.93-6553.46)	3561.58 (2413.23-5117.4)	33.54 (12.85-59.43)	25.56 (17.3-36.65)	-0.88 (-1.06, -0.64)
Kenya	3267.2 (2348.78-4255.53)	7905.07 (5728.02-10590.35)	48.7 (34.95-63.41)	46.28 (33.11-62.13)	-0.17 (-0.2, -0.13)
Kiribati	31.33 (24.12-39.08)	36.41 (26.68-50.05)	97.61 (74.12-122.14)	60.59 (44.96-81.03)	-1.57 (-1.64, -1.53)
Kuwait	78.57 (61.87-97.32)	255.67 (191.05-328.76)	17.15 (13.36-21.33)	11.37 (8.41-14.58)	-1.16 (-1.6, -0.76)
Kyrgyzstan	1772.25 (1225.62-2332.92)	1147.81 (821.42-1504.16)	66.02 (45.59-87.05)	27.16 (19.43-35.58)	-2.96 (-3.11, -2.8)
Lao People's Democratic Republic	2640.88 (2012.01-3366.81)	2934.48 (1772.68-3999.88)	148.22 (113.03-188.86)	76.93 (47.22-103.32)	-2.12 (-2.19, -2.08)
Latvia	1653.37 (849.83-2546.53)	450.67 (287.6-705.38)	47.09 (24.24-72.61)	9.46 (6.01-14.78)	-5.09 (-5.37, -4.73)
Lebanon	332.33 (198.27-507.68)	449.99 (288.35-660.15)	18.54 (11.21-28.1)	6.75 (4.34-9.91)	-3.25 (-3.42, -3.1)
Lesotho	468.64 (340.16-594.89)	732.06 (486.1-992.69)	64.51 (46.6-82.93)	82.92 (56.77-108.29)	0.9 (0.82, 0.98)

Liberia	812.4 (634.24-1000.75)	1246.08 (913.21-1695.07)	83 (65.41-101.55)	73.84 (55.33-99.89)	-0.38 (-0.47, -0.31)
Libya	309.32 (195.65-470.58)	855.12 (551.72-1257.22)	18.65 (11.86-28.56)	19.66 (12.78-28.77)	0.12 (-0.03, 0.27)
Lithuania	787.42 (384.41-1308.87)	294.31 (182.98-441.98)	17.6 (8.6-29.29)	4.34 (2.7-6.51)	-4.6 (-4.91, -4.28)
Luxembourg	83.02 (36.35-144.23)	14.53 (8.1-22.31)	15.69 (6.85-27.35)	1.17 (0.65-1.8)	-8.02 (-8.16, -7.91)
Madagascar	5520.35 (4504.8-6660.94)	8979.47 (6514.59-11861.85)	125.57 (102.44-151.56)	101.73 (74.79-132.99)	-0.68 (-0.73, -0.63)
Malawi	2541.09 (2013.17-3080.66)	4599.14 (3573.79-5757.32)	82.51 (65.19-100.11)	77.59 (60.38-97.73)	-0.16 (-0.2, -0.12)
Malaysia	2680.56 (1231.05-4591.83)	3022.71 (1959.09-4338.71)	31.83 (14.65-54.69)	12.28 (7.91-17.67)	-2.8 (-3.34, -2.12)
Maldives	67.74 (49.08-84.47)	20.72 (11.53-34.41)	94.06 (68.47-117.69)	7.41 (4.12-12.4)	-7.94 (-8.09, -7.82)
Mali	2377.62 (1812.04-2914.99)	4106.74 (3049.04-5343.82)	74.03 (56.07-90.91)	58.04 (44.03-74.04)	-0.75 (-0.78, -0.71)
Malta	45.46 (20.8-77.24)	22.59 (15.19-31.43)	11.54 (5.29-19.56)	1.99 (1.34-2.75)	-5.54 (-5.95, -5.3)
Marshall Islands	9.82 (6.55-13.37)	13.26 (8.41-20.06)	70.83 (47.98-96.66)	47.72 (30.86-70.81)	-1.42 (-1.51, -1.35)
Mauritania	822.76 (631.84-1054.25)	1073.61 (756.86-1509.48)	95 (73.05-121.26)	59.22 (42.42-82.66)	-1.53 (-1.59, -1.49)
Mauritius	93.81 (40.23-157.85)	62.07 (20.91-111.72)	14.82 (6.42-24.92)	3.64 (1.23-6.56)	-4.39 (-4.6, -4.18)
Mexico	5925.64 (3470.24-8406.15)	4611.92 (2932.22-7288.67)	17.34 (10.16-24.59)	3.97 (2.51-6.3)	-4.57 (-4.86, -4.42)
Micronesia (Federated States of)	45.22 (32.64-59)	29.19 (18.29-43.39)	104.43 (76.23-135.37)	48.18 (30.39-71.06)	-2.52 (-2.6, -2.48)

Monaco	5.56 (0.91-11.75)	3.01 (1.56-4.9)	6.62 (1.08-14.01)	2.39 (1.23-3.93)	-3.17 (-3.35, -2.99)
Mongolia	855.38 (628.97-1087.36)	722.05 (448.23-1081.3)	92.32 (67.9-116.24)	37.95 (23.31-56.89)	-3.28 (-3.53, -3.11)
Montenegro	294.23 (153.83-453.28)	314.12 (198.93-577.89)	51.38 (26.85-79.15)	37.19 (23.83-68.39)	-1.14 (-1.31, -1)
Morocco	4394.82 (2992.65-6265.62)	5934.18 (3915.7-8283.6)	35.3 (23.96-50.24)	20.61 (13.7-28.66)	-1.71 (-1.76, -1.67)
Mozambique	5200.27 (4119.56-6336.14)	10367.98 (7411.62-13314.13)	103.76 (82.54-125.71)	110.42 (80.64-142.49)	0.21 (0.17, 0.25)
Myanmar	29073.76 (22368.7-36888.77)	32999.32 (23053.82-44254.06)	144.02 (111.99-179.73)	77.72 (54.56-102.56)	-1.99 (-2.02, -1.96)
Namibia	385.67 (255.53-502.77)	392.64 (197.1-704.08)	78.2 (51.06-101.78)	37.11 (18.98-66.19)	-2.34 (-2.44, -2.29)
Nauru	0.36 (0-1.45)	0.4 (0.05-0.91)	9.2 (0-37)	7.96 (0.98-17.79)	-0.59 (-0.73, -0.43)
Nepal	4804.02 (3651.9-6156.42)	7990.49 (5893.66-10461.78)	62.34 (47.26-78.89)	40.12 (29.79-52.61)	-1.46 (-1.51, -1.4)
Netherlands	2268.76 (1134.35-3733.71)	905.66 (606.76-1269.26)	11.06 (5.53-18.21)	2.24 (1.5-3.13)	-5.09 (-5.25, -4.91)
New Zealand	80.91 (1.79-254.2)	93.15 (37.83-159.78)	2.14 (0.05-6.67)	0.99 (0.4-1.7)	-2.49 (-3.16, -1.91)
Nicaragua	367.95 (289.89-443.12)	446.28 (300.12-593.82)	27.18 (21.42-32.92)	10.29 (6.93-13.67)	-3.32 (-3.57, -3.16)
Niger	1672.22 (1227.22-2167.85)	4442.26 (3249.44-5938.66)	77.73 (57.18-99.86)	70.01 (51.92-93.69)	-0.31 (-0.35, -0.28)
Nigeria	30861.95 (23205.87-38814.1)	30925.57 (23241.52-40525.44)	83.21 (62.85-104.15)	43.55 (33-56.48)	-2.06 (-2.1, -2.02)
Niue	0.68 (0.22-1.19)	0.1 (0.01-0.25)	28.59 (9.37-49.96)	5.03 (0.5-11.97)	-5.52 (-5.61, -5.44)

North Macedonia	1629.9 (1038.3-2158.02)	1417.35 (1000.17-1971.25)	102.28 (65.2-134.95)	59.61 (42.75-82.43)	-1.81 (-1.93, -1.72)
Northern Mariana Islands	1.12 (0-2.98)	2.39 (1.13-4.18)	8.57 (0-23.23)	6.1 (2.88-10.84)	-1.22 (-1.55, -0.78)
Norway	447.13 (139.94-850.25)	67.58 (25.85-123.88)	5.78 (1.81-10.99)	0.55 (0.21-1.01)	-7.56 (-7.83, -7.33)
Oman	222.61 (146.28-316.66)	298.63 (203.8-416.47)	39.53 (26.13-55.69)	22.18 (15.25-31.08)	-1.92 (-2.07, -1.79)
Pakistan	25344.44 (19239.52-31478.83)	41409.73 (31075.5-54201.53)	51.48 (38.86-64.14)	41.34 (31.5-53.7)	-0.78 (-0.89, -0.7)
Palau	0.59 (0-1.6)	0.92 (0.09-1.94)	6.92 (0.03-18.88)	5.25 (0.5-11.09)	-0.84 (-1.09, -0.58)
Palestine	290.99 (201.59-413.64)	427.48 (283.68-575.97)	42.07 (29.18-59.04)	25.05 (16.51-33.96)	-2.15 (-2.32, -1.99)
Panama	234.14 (123.57-338.54)	164.01 (82.61-275.37)	16.98 (8.98-24.6)	3.59 (1.81-6.04)	-4.92 (-5.16, -4.76)
Papua New Guinea	1478.8 (1065.89-1948.24)	3141.69 (2213.29-4224.31)	105.69 (77.98-136.25)	80.14 (57.49-106.76)	-0.9 (-0.94, -0.86)
Paraguay	781.45 (457.47-1072.66)	523.15 (142.36-1049.29)	38.2 (22.29-52.45)	9.66 (2.64-19.39)	-4.54 (-4.75, -4.39)
Peru	3155.29 (2418.31-3892.87)	2435.17 (1509.96-3651.45)	27.97 (21.51-34.58)	7.25 (4.51-10.87)	-4.03 (-4.42, -3.68)
Philippines	13375.23 (10406.62-16370.14)	25698.75 (18005.82-34777.59)	58.27 (45.65-71.27)	35.61 (24.95-48.18)	-1.68 (-1.85, -1.48)
Poland	19324.19 (11876.56-26761.98)	6489.56 (4925.74-8817.25)	48.28 (29.54-66.94)	8.35 (6.34-11.34)	-5.68 (-5.86, -5.53)
Portugal	2816.06 (1084.3-5220.16)	567.4 (299.04-907.03)	22.73 (8.76-42.17)	1.79 (0.95-2.86)	-7.75 (-8.01, -7.52)
Puerto Rico	48.84 (0-158.22)	42.36 (10.85-80.4)	1.47 (0-4.76)	0.49 (0.13-0.92)	-3.3 (-3.58, -2.94)

Qatar	40.25 (30.49-50.78)	102.74 (73.26-138.1)	68.05 (52.29-85.82)	22.88 (16.17-29.53)	-3.56 (-3.96, -3.18)
Republic of Korea	9654.85 (3888.79-17031.36)	7195.02 (4678.85-10216.54)	45.24 (18.41-79.5)	7.93 (5.15-11.26)	-5.54 (-5.66, -5.39)
Republic of Moldova	2472.28 (1829-3103.31)	763.83 (487.06-1116.08)	68.15 (50.1-85.6)	12.63 (8.06-18.46)	-5.72 (-5.95, -5.51)
Romania	13340.64 (6854.86-19995.34)	5968.22 (4149.35-8976.17)	58.24 (29.94-87.18)	14.43 (10.05-21.67)	-4.47 (-4.61, -4.35)
Russian Federation	68309.93 (31813.38-108521.18)	24252.21 (14593.16-38197.88)	44.15 (20.55-70.27)	10.1 (6.08-15.89)	-4.69 (-5.01, -4.3)
Rwanda	3562.77 (2723.42-4568.36)	3181.79 (2228.51-4250.98)	149.56 (114.81-188.66)	65.36 (45.56-86.92)	-2.65 (-2.7, -2.6)
Saint Kitts and Nevis	6.37 (1.51-13.36)	2.56 (0.9-4.47)	17.69 (4.2-37.08)	4.78 (1.67-8.27)	-3.63 (-3.94, -3.19)
Saint Lucia	36.32 (18.61-57.95)	34.03 (14.29-59.61)	49.59 (25.32-78.78)	14.87 (6.23-26.09)	-3.91 (-4.11, -3.73)
Saint Vincent and the Grenadines	24.69 (14.11-38.87)	19.69 (7.51-36.07)	37.45 (21.3-58.94)	15.26 (5.81-27.98)	-2.75 (-2.95, -2.62)
Samoa	59.99 (44.07-76)	63.49 (37.96-89.01)	83.63 (62.1-106.68)	50.43 (29.87-70.08)	-1.65 (-1.72, -1.62)
San Marino	2.73 (1-5.06)	1.46 (0.73-2.45)	7.22 (2.65-13.37)	1.36 (0.67-2.28)	-5.54 (-5.88, -5.29)
Sao Tome and Principe	38.39 (30-46.17)	40.99 (30.24-53.59)	66.72 (52.58-80)	45.17 (33.82-58.61)	-1.21 (-1.29, -1.13)
Saudi Arabia	2288.65 (1586.66-3197.07)	4735.17 (3387.23-6191.95)	49.45 (34.54-68.61)	35.29 (25.85-45.28)	-1.19 (-1.3, -1.09)
Senegal	2279.26 (1792.63-2805.83)	4556.21 (3410.86-6038.34)	82.66 (65.43-101.22)	70.93 (53.06-93.33)	-0.53 (-0.67, -0.45)
Serbia	8219.76 (4580.57-11860.3)	5358.95 (3769.13-7806.32)	101.63 (57.3-146.98)	30.65 (21.54-44.58)	-3.76 (-3.97, -3.62)

Seychelles	4.15 (1.21-8.03)	4.43 (1.45-8.29)	7.41 (2.17-14.33)	4.34 (1.41-8.16)	-1.98 (-2.27, -1.72)
Sierra Leone	1553.5 (1202.11-1930.77)	2461.65 (1784.05-3176.64)	85.88 (66.96-105.8)	77.49 (57.46-99.01)	-0.34 (-0.37, -0.31)
Singapore	407.51 (147.83-704.16)	144.75 (79.13-229.69)	21.65 (7.85-37.29)	1.75 (0.96-2.78)	-7.85 (-8.2, -7.56)
Slovakia	1602.63 (837.44-2563.65)	719.82 (522.56-943.65)	27.86 (14.47-44.51)	7.51 (5.44-9.85)	-4.22 (-4.35, -4.07)
Slovenia	520.21 (268.45-818.65)	182.07 (126.7-256.57)	21.3 (11.01-33.47)	3.29 (2.29-4.66)	-6.12 (-6.33, -5.93)
Solomon Islands	133.66 (92.68-173.65)	301.67 (226.11-396.89)	129.03 (100.01-161.73)	109.1 (83.62-139.67)	-0.55 (-0.6, -0.5)
Somalia	2094.09 (1495.11-2780.95)	3661.47 (2497.79-5183.21)	109.36 (81.86-138.9)	76.72 (53.94-105.54)	-1.07 (-1.1, -1.05)
South Africa	4526.67 (3057.69-6159.97)	7612.36 (5255.32-10362.45)	24.45 (16.32-33.36)	19.9 (13.66-27.08)	-0.65 (-0.8, -0.5)
South Sudan	1796.34 (1289.61-2381.05)	1979.32 (1360.71-2763.86)	80.01 (58.11-104.23)	64.33 (45.6-87.13)	-0.7 (-0.74, -0.67)
Spain	6178.85 (2669.39-10820.92)	1725.46 (1055.19-2600.08)	11.7 (5.05-20.55)	1.3 (0.81-1.97)	-6.95 (-7.2, -6.63)
Sri Lanka	6562.85 (5360.43-7822.44)	6107.48 (2962.54-11312.98)	83.06 (67.94-98.89)	26.91 (13.13-49.94)	-3.75 (-3.92, -3.65)
Sudan	7418.07 (5421.33-9763.87)	7648.47 (5270.08-10592.04)	94.46 (68.86-124.2)	47.92 (32.97-65.03)	-2.3 (-2.37, -2.24)
Suriname	81.11 (36.89-134.62)	121.34 (54.79-207.29)	34.55 (15.75-57.21)	20.17 (9.09-34.49)	-1.67 (-1.89, -1.45)
Sweden	775.81 (204.57-1550.78)	145.75 (42.47-283.68)	4.55 (1.2-9.08)	0.52 (0.15-1.02)	-6.91 (-7.18, -6.67)
Switzerland	972.07 (445.61-1656.57)	230.59 (140.54-340.43)	8.36 (3.84-14.2)	0.95 (0.58-1.39)	-6.9 (-7.11, -6.78)

Syrian Arab Republic	1318.04 (877.42-1844.65)	2037.3 (1301.61-2946.49)	29.8 (19.63-41.45)	20.7 (13.46-29.28)	-1.22 (-1.38, -1.06)
Taiwan (Province of China)	3603.06 (1451.91-6666.27)	1761.69 (1309.56-2360.71)	27.85 (11.24-51.44)	4.06 (3.02-5.43)	-5.84 (-6.17, -5.52)
Tajikistan	1676.39 (1214.56-2184.65)	1954.68 (1376.6-2590.27)	67.64 (48.99-88.35)	45.84 (32.75-60.56)	-1.34 (-1.46, -1.2)
Thailand	15270.56 (11144.15-19292.11)	16309.83 (10735.29-23401.59)	51.44 (37.45-64.67)	15.22 (10.04-21.81)	-3.82 (-3.92, -3.72)
Timor-Leste	201.08 (152.87-250.42)	496.28 (278.71-709.13)	92.94 (71.58-114.92)	67.3 (37.65-96.16)	-1.05 (-1.12, -1)
Togo	882.45 (710.27-1078.62)	2396.38 (1762.91-3139.65)	87.74 (70.99-106.02)	81.29 (61.38-105)	-0.23 (-0.28, -0.19)
Tokelau	0.08 (0.01-0.3)	0.05 (0-0.12)	6.77 (0.41-23.86)	3.18 (0.03-8.14)	-2.43 (-2.56, -2.29)
Tonga	17.72 (12.85-22.67)	15.23 (8.03-22.64)	37.99 (27.69-48.68)	20.05 (10.57-29.77)	-2.12 (-2.22, -2.05)
Trinidad and Tobago	164.45 (29.17-374.48)	228.67 (75.37-432.91)	23.18 (4.09-52.68)	12.38 (4.08-23.41)	-2.06 (-2.23, -1.89)
Tunisia	938.95 (627.43-1360.12)	1652.92 (972.94-2441.55)	25.01 (16.67-36.16)	14.31 (8.33-21.12)	-1.83 (-1.9, -1.76)
Turkey	8125.56 (5508.05-11344.86)	10499.1 (7541.81-13773.62)	28.43 (19.21-39.56)	12.74 (9.15-16.66)	-2.59 (-2.75, -2.44)
Turkmenistan	313.16 (82.8-628.81)	852.01 (476.74-1382.48)	18.46 (4.92-36.91)	23.81 (13.49-38.47)	0.86 (0.56, 1.18)
Tuvalu	5.19 (3.87-6.74)	1.44 (0.82-2.17)	93.58 (69.73-119.45)	15.96 (9.17-24)	-5.61 (-5.73, -5.55)
Uganda	4295.14 (3215.83-5520.68)	6010.44 (4263.06-7940.45)	80.17 (59.77-101.76)	50.91 (36.51-67.52)	-1.49 (-1.52, -1.47)
Ukraine	30126.04 (15357.19-48133.32)	9912.35 (5668.02-16155.11)	46.47 (23.75-74.01)	12.53 (7.17-20.47)	-4.28 (-4.52, -4.02)

United Arab Emirates	123.5 (87.99-167.36)	354.48 (239.49-489.83)	40.54 (29.24-54.83)	25.55 (17.87-35.31)	-1.73 (-2.63, -0.92)
United Kingdom	10123.94 (4396.57-17313.68)	2309.5 (1474.04-3344.39)	10.59 (4.6-18.12)	1.48 (0.95-2.14)	-6.26 (-6.42, -6.11)
United Republic of Tanzania	6128.94 (4833.75-7704.51)	11007.9 (8053.76-14505.09)	67.79 (53.24-85.44)	53.89 (39.28-70.44)	-0.75 (-0.78, -0.72)
United States of America	14202.85 (5556.56-25175.79)	6262 (3122.39-10312.36)	4.26 (1.67-7.55)	0.99 (0.49-1.62)	-4.64 (-4.82, -4.43)
United States Virgin Islands	2.36 (0.05-6.45)	2.41 (1.08-4.17)	3.53 (0.08-9.6)	1.4 (0.62-2.41)	-2.78 (-3.02, -2.54)
Uruguay	527.71 (198.23-947.82)	224.93 (89.48-397.1)	13.82 (5.18-24.83)	3.45 (1.37-6.13)	-4.48 (-4.63, -4.31)
Uzbekistan	4517.33 (2859.02-6320.96)	5913.14 (4031.77-8109.36)	41.57 (26.29-58.21)	28.17 (19.45-38.58)	-1.39 (-1.57, -1.2)
Vanuatu	55.72 (42.42-71.56)	119.42 (90.01-151.72)	111.16 (84.88-140.13)	83.7 (63.47-104.88)	-0.95 (-0.98, -0.92)
Venezuela (Bolivarian Republic of)	1110.77 (497.77-1930.87)	1699.26 (904.17-2683.59)	12.83 (5.69-22.23)	6.08 (3.23-9.61)	-2.62 (-3.01, -2.12)
Viet Nam	41094.45 (30677-52134.65)	47270.35 (30711.78-63130.32)	114.08 (85.95-144.61)	55.81 (36.68-74.57)	-2.33 (-2.37, -2.29)
Yemen	3674.59 (2570.92-5090.15)	6232.39 (4131.38-9283.17)	95.03 (66.47-132.19)	56.64 (37.6-83.92)	-1.77 (-1.96, -1.61)
Zambia	2077.07 (1604.63-2580.96)	4110.66 (2865.03-5529.44)	90.88 (70.86-113.59)	76.87 (55.04-101.98)	-0.53 (-0.56, -0.5)
Zimbabwe	1615.37 (1255.89-1995.69)	3698.81 (2745.8-4806.47)	52.32 (40.54-64.7)	68.59 (51.97-87.27)	0.97 (0.91, 1.05)

ASMR: age-standardized mortality rate, AAPC: average annual percentage change, CI: confidence interval,SDI: sociodemographic index, UI: uncertainty interval.

Table 2: The DALYs cases and ASDR of stroke attributable to air pollution in 1990 and 2021 and its trends.

	DALYs				
	1990 counts (95%UI)	2021 counts (95%UI)	1990 ASR (95%UI)	2021ASR (95%UI)	AAPC (95%CI)
Global	42304117.5 (34553909.64-49981909.54)	44962166.97 (35020338.76-55467023.53)	1073.52 (877.41-1276.32)	523.3 (407.96-645.58)	-2.42 (-2.47, -2.36)
Sex					
Male	22042113.99 (17840356.68-26509029.51)	24804489.97 (18892806.31-31076582.44)	1211.35 (978.24-1459.82)	619.37 (471.91-777.85)	-2.21 (-2.31, -2.15)
Female	20262003.51 (16469195.78-24428886.13)	20157677.01 (15842114.84-25207194.65)	955.8 (776.39-1152.5)	439 (345.08-549.03)	-2.6 (-2.66, -2.54)
SDI					
High SDI	2802117.29 (1970060.92-3869337.02)	1625946.6 (1232130.37-2082328.67)	254.27 (178.36-350.52)	80.32 (61.68-102.39)	-3.68 (-3.74, -3.64)
High-middle SDI	10874231.62 (8518190.42-13473283.43)	7801888.17 (5931357.33-10188263.45)	1133.11 (886.3-1405.11)	398.87 (303.25-520.76)	-3.36 (-3.48, -3.25)
Middle SDI	16139883.95 (13229208.13-19323108.98)	16823973.99 (12428844.31-21838218.68)	1617.52 (1322.7-1942.01)	645.43 (478.24-838.92)	-2.97 (-3.14, -2.89)
Low-middle SDI	8677233 (7121160.17-10185553.84)	12835904.8 (10109818.69-15248022.76)	1422.35 (1162.67-1675.4)	899.28 (707.45-1071.12)	-1.49 (-1.54, -1.42)
Low SDI	3768690.01 (3076522.18-4476852.89)	5841672.12 (4664357.38-6927648.54)	1680.76 (1375.99-1984.48)	1162.18 (934.76-1380.38)	-1.2 (-1.22, -1.17)
21 GBD Region					
Andean Latin America	153317.66 (119191.68-192516.81)	107297.23 (69414.88-155396.18)	714.26 (556.37-894.71)	179.01 (116.19-259.78)	-4.38 (-4.51, -4.27)
Australasia	9918.31 (333.26-28687.77)	12482.13 (7290.18-18772.77)	43.18 (1.46-125.15)	22.15 (13.11-33.35)	-2.1 (-2.61, -0.77)

Caribbean	178317.47 (128648.56-237651.9)	225301.45 (161358.27-303806.82)	679.42 (489.72-909.71)	422.11 (302.37-568.58)	-1.47 (-1.53,-1.43)
Central Asia	409971.23 (235785.55-590567.55)	431400.51 (321572.62-557624.02)	888.7 (511.59-1285.53)	551.05 (409.13-711.03)	-1.56 (-1.72,-1.42)
Central Europe	1360957.8 (802426.33-1926982.09)	526957.72 (387495.29-764557.02)	954.98 (563.1-1349.13)	232.35 (170.66-337.76)	-4.54 (-4.64,-4.46)
Central Latin America	321344.48 (214343.15-443186.23)	288131.77 (197134.14-408309.03)	384.85 (256.36-531.99)	115.57 (79.06-163.68)	-3.8 (-3.9, -3.72)
Central Sub-Saharan Africa	374669.25 (277135.64-477301.92)	654074.68 (469592.46-872235.9)	1743.43 (1292.2-2209.99)	1272.78 (919.31-1708.91)	-0.99 (-1.07,-0.94)
East Asia	17984928.61 (14430399.56-22079588.07)	16774343.34 (12321391.38-22074556.32)	2232.68 (1783.44-2718.4)	796.84 (587.17-1047.9)	-3.33 (-3.46,-3.25)
Eastern Europe	2139593.01 (1090190.43-3324697.58)	716463.6 (446762.09-1081736.56)	802.65 (408.75-1245.24)	206.78 (128.93-312.43)	-4.26 (-4.5, -4.01)
Eastern Sub-Saharan Africa	1470327.64 (1195081.86-1782866.11)	2131931.03 (1687519.05-2583390.08)	1966.06 (1599.36-2375.57)	1273.51 (1009.75-1537.19)	-1.39 (-1.41,-1.37)
High-income Asia Pacific	488978.28 (149575.24-940155.03)	372191.3 (223080.36-547976.67)	250.24 (75.72-482.94)	80.55 (48.96-118.07)	-3.57 (-3.67,-3.45)
High-income North America	310232.72 (121162.91-535705.68)	134565.16 (65311.55-218594.47)	87.86 (34.34-151.65)	20.89 (10.16-33.88)	-4.6 (-4.76,-4.39)
North Africa and Middle East	1629549.86 (1267034.06-2006930.55)	2369293.12 (1807897.36-2972673.98)	1005.92 (783.21-1239)	537.1 (411.16-667.99)	-2.11 (-2.2, -2.02)
Oceania	61788.57 (45017.5-80334.89)	114273 (82842.61-151174.36)	2056.51 (1537.83-2653.98)	1485.18 (1081.84-1941.14)	-1.08 (-1.11, -1.05)
South Asia	6698028.85 (5379938.58-7941527.22)	11015792.37 (8784786.02-13085043.78)	1163.61 (932.62-1382.07)	750.84 (599.66-893.92)	-1.46 (-1.57,-1.37)
Southeast Asia	4940642.84 (3943587.61-5866476.94)	5692351.34 (3958956.38-7553310.93)	1937.1 (1531.47-2322.1)	879.64 (612.33-1165.29)	-2.57 (-2.67,-2.52)

Southern Latin America	187184.58 (97917.27-288581.28)	89749.55 (53911.91-137512.2)	412.49 (215.95-636.83)	103.06 (61.87-158.01)	-4.37 (-4.5, -4.28)
Southern Sub-Saharan Africa	195643.41 (146478.96-250487.26)	316348.63 (232582.9-412506.55)	715.78 (533.84-917.53)	562.04 (415.33-733.89)	-0.81 (-0.91, -0.72)
Tropical Latin America	578570 (337772.63-878377.36)	294394.14 (170420.6-448921.31)	630.67 (372.26-951.5)	114.93 (66.55-175.36)	-5.4 (-5.51, -5.31)
Western Europe	1292282.33 (626722.02-2164966.74)	359395.76 (245095.63-499981.38)	219.47 (106.4-367.09)	34.99 (23.77-48.5)	-5.82 (-5.92, -5.74)
Western Sub-Saharan Africa	1517870.6 (1210154.4-1819258.24)	2335429.15 (1803109.57-2892797.67)	1767.74 (1413.99-2114.75)	1213.21 (941.52-1490.9)	-1.21 (-1.23, -1.19)
204 Countries and Regions					
Afghanistan	182624.68 (130109.18-239970.38)	186220.3 (133033.57-248458.22)	2731.97 (1970.02-3553.83)	1809.83 (1300.76-2374.6)	-1.34 (-1.4, -1.29)
Albania	26380.68 (19876.39-32853.9)	15005.56 (8847.98-24907.59)	1426.37 (1077.71-1772.04)	354.82 (209.47-587.9)	-4.54 (-4.76, -4.36)
Algeria	51697.86 (31771.29-73573.55)	99887.64 (64645.37-145270.47)	505.63 (317.9-717.22)	323.46 (210.5-469.41)	-1.3 (-1.38, -1.22)
American Samoa	29.62 (0-118.52)	51.26 (4.29-130.42)	129.44 (0-519.85)	109.99 (9.22-279.38)	-0.44 (-0.53, -0.36)
Andorra	49.37 (20.06-92.91)	32.07 (16.91-49.1)	91.91 (37.58-172.15)	19.9 (10.49-30.74)	-4.86 (-5.01, -4.75)
Angola	71551.72 (52427.79-93100.7)	92559.72 (56971.1-128831.3)	1898.26 (1416.29-2474.99)	842.92 (535.52-1172.93)	-2.6 (-2.72, -2.51)
Antigua and Barbuda	187.74 (42.8-433.05)	191.46 (68.2-345.29)	345.18 (78.68-797.85)	186.81 (66.98-338.1)	-1.98 (-2.15, -1.8)
Argentina	120908.84 (55124.32-199720.01)	51284.81 (26318.67-84931.75)	383.26 (174.93-633.1)	91.91 (47.11-152.14)	-4.46 (-4.61, -4.33)

Armenia	15824.27（9314.38-23988.28)	13400.56（9014.99-18615.37)	618.39（364.63-939.48)	312.81 (210.52-434.13)	-2.18 (-2.37,-1.96)
Australia	8385.82（273.7-24018.58)	10929.53（6392.36-16344.73)	43.93（1.44-126.07)	22.94（13.45-34.18)	-2.01 (-2.5, -1.29)
Austria	31907.98（16504.58-51440.7)	6973.84（4798.61-9495.14)	261.41（134.77-421.06)	35.43（24.57-48.07)	-6.35 (-6.47,-6.22)
Azerbaijan	33110.29 (13794.73-53208.52)	31120.53（16289.31-50866.1)	680.95 (282.25-1096.66)	335.15（177.7-553.4)	-2.29 (-2.44,-2.19)
Bahamas	428.48（66.74-989.63)	647.2（201.83-1300.96)	269.31（42.05-620.79)	160.69（50.32-323.74)	-1.65 (-1.82,-1.48)
Bahrain	1683（1299.83-2096.34)	3487.12（2591.03-4443.93)	1105.12 (850.5-1391.81)	491.74 (363.17-620.42)	-2.71 (-2.85,-2.59)
Bangladesh	1067297.25 (849311.71-1309766.94)	1808652.1（1357057.5-2330204.51)	2228.6 (1788.93-2730.67)	1357.16 (1019.04-1740.14)	-1.57 (-1.7, -1.39)
Barbados	1045.44（271.82-2278.05)	1136.29（463.62-2011.97)	356.31（92.69-777.3)	223.73（91.38-395.02)	-1.47 (-1.64,-1.26)
Belarus	85667.13 (45533.53-131539.53)	36142.33（24645.31-49440.15)	671.52 (357.13-1031.38)	227.65 (155.16-311.35)	-3.6 (-3.81,-3.33)
Belgium	36140.47 (18205.85-59816.26)	9078.24（6135.37-12297.28)	233.54（117.71-386.84)	36.02（24.73-48.47)	-5.92 (-6.02, -5.8)
Belize	412.58（240.9-637.36)	735（341.92-1200.42)	433.45（252.83-668.69)	245.37 (113.44-400.72)	-1.76 (-1.96, -1.6)
Benin	39327.07（31463.1-47920.68)	73169.89（54963.49-92799.5)	2025.67 (1623.04-2459.08)	1484.02 (1125.27-1874.09)	-1.02 (-1.06,-0.98)
Bermuda	44.77（0-120.64)	26.38（4.91-52.38)	73.93（0-198.84)	19.01（3.53-37.9)	-4.27 (-4.34,-4.17)
Bhutan	2591.74（1838.99-3465.33)	2274.07（1517.99-3159.36)	1086.31 (772.76-1456.49)	381.84 (256.11-527.82)	-3.65 (-3.95 -3.44)
Bolivia (Plurinational State of)	41142.47 (28720.33-57305.46)	29973.96（18626.74-46921.06)	1242.41 (869.24-1727.17)	332.1（207.52-521.16)	-4.33 (-4.4, -4.25)

Bosnia and Herzegovina	54190. 72 (43647. 45-65306. 85)	31843. 61 (21280. 14-49915. 48)	1449. 43 (1164. 1-1753. 37)	504. 71 (337. 01-790. 27)	-3. 39 (-3. 49, -3. 3)
Botswana	7893. 54 (4988. 86-10805. 12)	5093. 81 (2688. 76-9039. 8)	1513. 82 (962. 78-2066. 99)	379. 89 (202. 5-673. 9)	-4. 67 (-4. 9, -4. 52)
Brazil	561167. 21 (325161. 93-854483. 91)	283107. 62 (165128. 66-429304. 34)	626. 45 (366. 94-948. 69)	113. 11 (66. 03-171. 6)	-5. 42 (-5. 54, -5. 33)
Brunei Darussalam	149. 65 (9. 83-385. 46)	197. 27 (41. 76-398. 9)	140. 71 (9. 27-365. 38)	59. 33 (12. 63-119. 33)	-2. 88 (-3. 12, -2. 69)
Bulgaria	139667. 01 (76122. 84-205109. 33)	66758. 36 (47624. 14-102301. 42)	1259. 16 (686. 79-1849. 28)	468. 91 (333. 92-720. 14)	-3. 25 (-3. 41, -3. 06)
Burkina Faso	56153. 04 (43879. 2-70515. 26)	103174. 95 (76608. 64-133153. 67)	1324. 43 (1040. 07-1655. 61)	1127. 33 (842. 25-1428. 14)	-0. 51 (-0. 54, -0. 48)
Burundi	70109. 45 (51205. 69-90594. 29)	73904. 17 (54645. 06-95487. 43)	2973. 58 (2184. 74-3810. 83)	1521. 02 (1134. 48-1977. 23)	-2. 16 (-2. 19, -2. 13)
Cabo Verde	2319. 78 (1796. 17-2863. 65)	2951. 16 (2053. 86-3931. 9)	1020. 68 (787. 43-1262. 08)	670. 52 (468. 2-894. 79)	-1. 25 (-1. 35, -1. 15)
Cambodia	115510. 13 (91560. 46-139219. 22)	191381. 62 (141622. 02-244941. 48)	2595. 66 (2064. 39-3125. 97)	1645. 04 (1238. 53-2068. 23)	-1. 46 (-1. 49, -1. 44)
Cameroon	72270. 55 (54386. 97-93051. 34)	179776. 62 (127217. 97-247250. 1)	1646. 29 (1248. 62-2102. 79)	1432. 29 (1030. 24-1945. 57)	-0. 44 (-0. 49, -0. 41)
Canada	19225. 87 (5396. 86-36348. 61)	7828. 77 (2931. 58-14306. 07)	59. 76 (16. 79-112. 9)	10. 75 (4. 05-19. 57)	-5. 34 (-5. 55, -5. 11)
Central African Republic	28131. 17 (19950. 69-37399. 62)	45402. 23 (30670-62504. 93)	2517. 78 (1805. 98-3274. 95)	2103. 13 (1459. 55-2879. 41)	-0. 55 (-0. 59, -0. 53)
Chad	47668. 18 (36777. 92-59381. 75)	100395. 07 (73952. 7-132962. 18)	1709. 95 (1316. 68-2119. 55)	1740. 11 (1289. 98-2280. 5)	0. 06 (0. 02, 0. 1)
Chile	55456. 55 (38122. 58-74925. 95)	34489. 15 (22518. 52-49018. 5)	562. 59 (387. 04-760. 64)	135. 03 (88. 13-191. 62)	-4. 59 (-4. 67, -4. 51)
China	17531913. 93 (14018799. 2-21553686. 3)	16063167. 91 (11839650. 6-21366582. 1)	2265. 23 (1809. 67-2761. 38)	792. 49 (585. 1-1051. 35)	-3. 39 (-3. 53, -3. 31)

Colombia	80494.13 (49934.39-111454.66)	41163.35（24931.59-64107.65)	451.68（280.99-625.77)	74.48（45.07-115.98)	-5.74 (-5.92, -5.62)
Comoros	3853.83（2758.51-4950.15)	5038.86（3534.66-6586.32)	1983.48 (1456.29-2511.47)	1064.38 (753.72-1379.26)	-2.01 (-2.08, -1.94)
Congo	22691.74 (16266.46-29522.05)	28864.99（19391.22-40125.45)	2206.62 (1611.87-2836.31)	1123.1 (763.81-1506.61)	-2.12 (-2.22, -2.05)
Cook Islands	19.43（0.65-58.53)	10.17（0-28.25)	157.25（5.3-472.82)	40.95（0-113.5)	-4.19 (-4.3, -4.1)
Costa Rica	3915.95（2097.85-5648.06)	3086.93（1928.24-4586.83)	221.91（119.15-320.6)	56.05（35-83.36)	-4.57 (-4.76, -4.39)
Coted'Ivoire	70372.63 (53759.56-90705.18)	156901.74（111862.93-211662.34)	1748.35 (1357.03-2209.7)	1396.87 (1016.82-1866.18)	-0.71 (-0.77, -0.66)
Croatia	39074.12 (20097.48-62048.51)	12834.41（9209.64-16919.99)	696.08 (358.04-1103.59)	135.5（97.06-178.32)	-5.17 (-5.33, -4.93)
Cuba	27150.65 (10354.69-53179.72)	32279.96（14892.62-54505)	268.15（102.27-525.3)	164.49（76.03-277.75)	-1.43 (-1.63, -1.27)
Cyprus	2100.54（929.56-3685.73)	1305.16（896.68-1742.56)	351.97（159.04-616.85)	71.99（49.44-96.27)	-5.26 (-5.45, -4.83)
Czechia	90226.04 (47131.24-146039.49)	17608.73（12700.06-23387.16)	655.91（343.17-1062)	79.92（57.32-106.11)	-6.58 (-6.76, -6.44)
Democratic People's Republic of Korea	363005.51 (269259.06-477588.86)	667531.5（510679.27-859343.46)	2338.99 (1746.08-2997.95)	2038.6 (1568.5-2624.72)	-0.44 (-0.47, -0.43)
Democratic Republic of the Congo	243508.02 (172210.72-315226.94)	479998.21（334621.48-665617.55)	1652.08 (1195.35-2139.82)	1406.99 (992.34-1940.78)	-0.54 (-0.61, -0.48)
Denmark	15405.84（6842.43-26532.3)	3937.81（2348.16-5818.68)	185.43（82.31-319.22)	31.46（18.77-46.32)	-5.74 (-5.89, -5.57)
Djibouti	1910.72（1271.17-2683.43)	5151.32（3214.01-7593.2)	1412.84 (954.28-1927.29)	835.47 (522.1-1234.18)	-1.78 (-1.87, -1.7)

Dominica	414.11（236.9-604.19）	225.4（102.49-392.51）	704.91（400.83-1024.37）	278.63（126.5-483.29）	-2.94（-3.01，-2.9）
Dominican Republic	23432.87（14437.09-32731.32）	24486.13（8886.86-44649.15）	623.53（385.89-869.4）	242.31（87.87-440.34）	-2.94（-3.07,-2.86）
Ecuador	32600.12（22024.28-43661.23）	19488.66（11405.16-29826.76）	584.99（395.7-781.98）	120.11（70.4-183.71）	-5.04（-5.24,-4.81）
Egypt	419027.29（312624.33-542051.98）	667456.58（461882.15-871252.74）	1770.72（1327.07-2290.53）	1224.51（867.51-1589.76）	-1.31（-1.48,-1.19）
El Salvador	18228.4（14509.47-22178.78）	8161.74（4888.15-11985.77）	593.19（474.39-721.66）	129.49（77.46-190.64）	-4.77（-4.97,-4.58）
Equatorial Guinea	4255.26（3073.42-5518.11）	2387.08（1139.33-4073.56）	2249.15（1646.69-2867.05）	498.82（242.46-834.23）	-4.68（-4.82,-4.57）
Eritrea	31693.43（23352.29-41273.54）	42090.53（29531.49-55400.4）	2660.29（1973.54-3402.3）	1520.9（1077.12-1972.53）	-1.85（-1.9，-1.81）
Estonia	8858.72（3455.99-16000.37）	650.12（207.71-1387.4）	436.49（169.72-787.18）	23.57（7.6-49.81）	-9.27（-9.6，-8.99）
Eswatini	4273.84（3062.25-5646.74）	5226.83（2649.96-8774.69）	1594.12（1143.14-2089.81）	979.96（514.82-1605.07）	-1.65（-1.71,-1.59）
Ethiopia	400722.42（312896.48-539034.42）	362327.04（284793.78-450946.92）	1971.26（1557.56-2562.52）	839.02（655.76-1043.7）	-2.75（-2.79,-2.72）
Fiji	4689.76（2746.25-6469.43）	3372.33（1022.5-6359.8）	1248.09（730.85-1704.58）	461.37（142.32-867.87）	-3.34（-3.46,-3.24）
Finland	6008.7（942.86-12597.94）	1243.8（172.24-2803.61）	84.06（13.21-176.47）	9.26（1.29-20.79）	-7.27（-7.72,-6.96）
France	123125.3（56775.9-211811.1）	39884.38（25869.19-57112.28）	144.02（66.16-247.63）	25.49（16.58-36.47）	-5.39（-5.49 -5.31）
Gabon	4531.36（2374.41-7355.68）	4862.45（2736.31-7743.82）	822.44（431.93-1342.94）	499.86（280.07-792.36）	-1.45（-1.56,-1.34）
Gambia	6408.81（4804.75-8196.44）	17382.61（12497.26-22716.55）	1822.57（1388.12-2316.05）	1791.24（1304-2310.47）	0.05（-0.07，0.19）

Georgia	50217.86 (18647.16-84764.38)	31942.69（19225.98-48941.45)	825.47 (306.99-1388.88)	526.09 (316.81-807.83)	-1.79 (-1.99,-1.58)
Germany	338167.18 (166729.05-563358.84)	77116.52（51642.51-107300.73)	259.45（127.81-433.24)	37.47（25.12-51.88)	-6.11 (-6.27 -5.95)
Ghana	146595.76 (113208.39-181853.92)	300336.35（214884.97-383005.34)	2295.02 (1771.73-2829.47)	1796.67 (1314.08-2279.32)	-0.79 (-0.83 -0.74)
Greece	64293.05 (32565.86-105103.29)	26187.53（18719.67-34455.04)	434.73（219.97-710.08)	94.84（68.18-124.78)	-4.82 (-4.97,-4.67)
Greenland	40.42（0.21-110.83)	22.66（0.86-60.67)	127.33（0.62-348.54)	36.41（1.39-97.39)	-3.77 (-3.93, -3.6)
Grenada	649.63（360.32-1018.82)	326.62（130.13-602.59)	902.34 (500.67-1409.53)	301.87 (120.97-553.58)	-3.61 (-3.79,-3.49)
Guam	74.58（0-211.09)	145.38（66.83-236.93)	101.89（0-286.82)	71.67（33.15-116.97)	-0.94 (-1.3, -0.32)
Guatemala	21500.56 (17134.64-25770.32)	29284.11（17515.85-41498.92)	615.76（491.02-736.05)	267.46 (160.51-379.58)	-2.73 (-3.03,-2.51)
Guinea	58196.45 (44597.34-71690.14)	94749.44（69873.37-123521.21)	1779.5 (1363.15-2188.83)	1689.4 (1261.42-2191.89)	-0.17 (-0.2, -0.15)
Guinea-Bissau	12323.68（9295.26-16172.84)	18299.79（13266.2-23928.22)	3023.76 (2278.28-3900.49)	2448.71 (1793.1-3112.93)	-0.66 (-0.69,-0.64)
Guyana	6098.97（3473.72-9579.95)	3677.47（1629.25-6340.25)	1575.87 (899.41-2472.8)	582.61 (257.76-996.51)	-3.2 (-3.36,-2.89)
Haiti	91061.47 (70822.33-115087.94)	136199.77（95391.58-188879.96)	2815.75 (2188.89-3537.43)	1879.27 (1316.91-2593.86)	-1.29 (-1.33,-1.27)
Honduras	20229.61（15588.8-24905.82)	48520.59（33261.89-64832.02)	932.03 (719.94-1136.66)	783.98 (538.69-1030.85)	-0.56 (-0.69,-0.44)
Hungary	112406.24 (59120.65-181450.68)	24248.49（16936.73-41012.45)	790.38 (415.94-1276.31)	123.26（85.85-207.5)	-5.93 (-6.08,-5.76)
Iceland	121.14（9.16-288.89)	39.98（7.44-88.78)	40.99（3.1-97.93)	6.45（1.19-14.28)	-6.09 (-6.38,-5.79)

India	4877500.73 (3859662.18-5874901.43)	7917327.48 (6340815.49-9592697.55)	1040.91 (821.86-1253.39)	661.89 (531.49-803.23)	-1.5 (-1.62, -1.4)
Indonesia	2037499.34 (1584879.33-2498786.13)	2249839.73 (1459023.24-3214391.41)	2032 (1579.98-2517.25)	975.44 (637.51-1376.04)	-2.42 (-2.57,-2.35)
Iran (Islamic Republic of)	138265.27 (107709.62-170460.97)	210861.39 (165108.5-263855.74)	596.56 (463.26-735.04)	285.77 (224.07-358.04)	-2.41 (-2.54, -2.3)
Iraq	106960.16 (74379.12-144823.33)	192379.44 (127645.15-270466.1)	1322.22 (916.84-1780.64)	861.04 (574.84-1192.06)	-1.29 (-1.36,-1.22)
Ireland	5811.77 (2291.26-10507.56)	1259.65 (649.09-2012.84)	144.22 (56.9-261.02)	15.53 (7.98-24.81)	-7.02 (-7.14,-6.88)
Israel	10066.5 (5388.17-16130.28)	7762.87 (5918.14-9969.59)	211.76 (113.59-338.78)	60.57 (46.17-78.11)	-4.04 (-4.42,-3.48)
Italy	253134.66 (136956.52-393135.84)	87049.52 (63042.89-115733.8)	287.3 (155.56-446.69)	51.26 (37.51-67.41)	-5.55 (-5.69,-5.38)
Jamaica	13414.08 (8011.05-18266.42)	8312.46 (4459.49-13062.83)	744.85 (444.89-1013.4)	264.13 (141.8-415.28)	-2.95 (-3.66,-2.42)
Japan	239466.01 (46730.56-515149.52)	220138.28 (121100.33-339439.71)	147.26 (28.73-316.93)	59.96 (32.78-91.55)	-2.78 (-2.88,-2.62)
Jordan	6409.39 (4687.44-8362.09)	20045.48 (14266.17-26252.72)	512.48 (373.92-669.75)	300.66 (215.89-390.63)	-1.81 (-2.01,-1.68)
Kazakhstan	85499.01 (32407.91-150932.37)	79183.4 (53752.42-114057.73)	700.66 (265.97-1238.43)	482.58 (328.54-687.4)	-1.18 (-1.31,-1.01)
Kenya	80715.25 (59635.26-104531.48)	198262.89 (147488.31-262317.68)	1021.41 (753-1316.59)	932.21 (687.96-1229.01)	-0.32 (-0.34,-0.29)
Kiribati	983.78 (767.99-1230.52)	1157.59 (844.22-1578.4)	2477.24 (1921.37-3095.61)	1487.45 (1095.13-2036.59)	-1.68 (-1.75,-1.64)
Kuwait	2482.87 (1953.14-3092.01)	7362.32 (5491.97-9338.79)	375.41 (295.66-464.6)	238.48 (179.28-301.1)	-1.71 (-2.12,-1.17)
Kyrgyzstan	41163.33 (28152.4-53942.95)	31145.93 (22453.39-40722.18)	1412.02 (969.84-1852.23)	633.79 (455.81-826.71)	-2.63 (-2.84,-2.48)

Lao People's Democratic Republic	73080.76 (56002.09-93735.24)	76900.5 (46001.33-104845.35)	3457.62 (2647.16-4392.37)	1659.52 (998.55-2238.23)	-2.37 (-2.42, -2.33)
Latvia	30978.42 (15828.55-47970.23)	7226.21 (4648.51-11335.43)	868.82 (444.4-1345.75)	169.22 (108.7-264.47)	-5.2 (-5.48, -4.85)
Lebanon	7724.88 (4660.89-11729.74)	8477.37 (5407.35-12365.99)	373.26 (224.83-569.48)	134.2 (85.58-195.54)	-3.29 (-3.46, -3.13)
Lesotho	10527.93 (7675.25-13322.17)	18249.68 (11946.7-25130.83)	1304.82 (954.93-1649.88)	1755.32 (1173.13-2383.22)	1.01 (0.91, 1.08)
Liberia	20135.96 (15736.26-24982.77)	33395.61 (24382.04-45140.79)	1779.99 (1404.29-2194.17)	1551.28 (1149.88-2087.08)	-0.44 (-0.5, -0.38)
Libya	7360.19 (4786.23-11004.11)	21652.47 (13908.45-31434.04)	386.5 (249.93-578.29)	408.81 (265.65-595.5)	0.13 (-0.01, 0.27)
Lithuania	16616.01 (8172.06-27248.05)	5022.7 (3129-7470.69)	370.92 (182.4-607.88)	84.22 (52.33-125.07)	-4.76 (-4.99, -4.46)
Luxembourg	1391.27 (601.49-2433.18)	231.15 (128.47-355.78)	257.17 (110.86-449.66)	20.43 (11.41-31.49)	-7.9 (-8.03, -7.8)
Madagascar	149471.39 (122376.79-182122.65)	263339.43 (192659.08-346560.31)	2878.88 (2358.52-3492.59)	2265.79 (1669.81-2952.31)	-0.77 (-0.82, -0.71)
Malawi	67091.6 (53492.83-81475.63)	120268.25 (93130.62-150447.3)	1790.25 (1430.15-2163.48)	1648.04 (1283.99-2060.12)	-0.24 (-0.27, -0.19)
Malaysia	69837.74 (31823.05-120527.79)	75981.73 (50271.92-108378.05)	741.64 (338.18-1284.62)	272.4 (178.76-389.41)	-3.04 (-3.37, -2.71)
Maldives	2012.32 (1448.51-2561.24)	503.58 (279.29-828.62)	2182.57 (1593.5-2737.97)	145.46 (81.07-240.82)	-8.48 (-8.6, -8.39)
Mali	64511.47 (49566.55-79399.96)	108564.04 (81120.14-142997.05)	1638.3 (1264.41-2004.55)	1240.39 (935.74-1615.24)	-0.88 (-0.91, -0.86)
Malta	850.07 (387.16-1450.42)	373.92 (258.44-519.32)	204.63 (93.26-349.26)	37.07 (25.67-51.43)	-5.56 (-5.83, -5.34)
Marshall Islands	282.65 (188.09-384.63)	423.8 (271.32-646.98)	1640.97 (1102.83-2220.23)	1131.03 (725.63-1677.74)	-1.34 (-1.43, -1.27)

Mauritania	20487.6（15830.08-25945.27）	25188.49（17657.14-35302.7）	2075.28（1603.73-2637.08）	1205.65（856.7-1675.79）	-1.78（-1.83,-1.74）
Mauritius	2475.36（1062.23-4133.31）	1521.54（514.5-2749.14）	337.74（146.43-564.91）	86.51（29.3-156.46）	-4.21（-4.43,-3.98）
Mexico	135262.41（79784.1-194441.83）	106309.05（68078.43-166785.14）	329.72（194.26-472.32）	85.02（54.29-133.48）	-4.22（-4.36,-4.11）
Micronesia (Federated States of)	1260.39（911.66-1645.57）	874.24（541.6-1309.37）	2487.45（1796.24-3238.54）	1146.34（720.04-1682.44）	-2.52（-2.6, -2.48）
Monaco	85.28（13.66-179.31）	44.89（23.3-73.4）	112.66（17.93-235.32）	42.24（21.76-70.13）	-3.06（-3.21, -2.9）
Mongolia	21479.39（15953.63-27366.62）	20091.85（12383.89-29588.72）	2058.79（1528.75-2611.94）	850.14（525.08-1254.29）	-3.07（-3.34,-2.88）
Montenegro	5743.57（3026.17-8837.67）	5216.72（3334.46-9630.9）	951.56（500.87-1463.68）	574.24（367.83-1060.38）	-1.63（-1.74,-1.54）
Morocco	101924.21（68980.56-144538.25）	129260.46（85367.7-184111.19）	723.9（493.05-1028.09）	395.93（263.94-557.45）	-1.92（-1.97,-1.88）
Mozambique	135422.59（108294.47-164610.49）	284681.43（202315.4-367414.96）	2295.75（1832.81-2785.22）	2501.53（1791.02-3209.44）	0.28（0.25, 0.31）
Myanmar	799582.8（613361.86-1018669.56）	815831.68（573089.25-1097155.52）	3377.7（2606.82-4265.79）	1690.46（1189.43-2265.99）	-2.23（-2.27, -2.2）
Namibia	9602.33（6314.78-12561.69）	9231.43（4595.48-16523.22）	1599.65（1060.06-2076.88）	730.77（367.87-1305.84）	-2.48（-2.59,-2.42）
Nauru	11.58（0-46.85）	12.65（1.46-28.87）	226.8（0-913.02）	197.05（23.32-445.59）	-0.57（-0.72,-0.41）
Nepal	126635.1（95512.3-161047.49）	187026.51（138220.37-243070.85）	1368.94（1046.27-1739.61）	826.59（612.62-1075.15）	-1.66（-1.71, -1.6）
Netherlands	41424.74（20200.44-68805.71）	14529.02（9917.53-20126）	204.77（100.06-339.87）	39.24（26.85-54.28）	-5.28（-5.44,-5.08）
New Zealand	1532.49（36.49-4843.55）	1552.6（633.36-2592.53）	39.54（0.95-124.97）	17.9（7.33-29.84）	-2.46（-3.05, -1.9）

Nicaragua	9144.33（7206.08-10899.38）	10729.75（7308.29-14218.39）	582.71（458.18-696.07）	220.45（151.39-292.98）	-3.29（-3.52,-3.11）
Niger	46029.53（33815.41-59452.1）	114309.47（83581.62-151440.58）	1690.11（1254.97-2175.61）	1447.38（1070.24-1910.38）	-0.49（-0.52,-0.46）
Nigeria	733377.13（555231.22-924984.53）	766297.57（568581.1-1009910.04）	1722.65（1307.84-2160.48）	883.79（669.78-1154.05）	-2.13（-2.17,-2.09）
Niue	14.12（4.63-25.02）	2.34（0.23-5.63）	633.69（207.48-1127.05）	111.85（10.94-267.55）	-5.52（-5.59,-5.45）
North Macedonia	32958.66（20936.12-43596.39）	25548.93（17954.61-35616.17）	1901.03（1205.78-2520.95）	909.58（644.7-1272.01）	-2.42（-2.55,-2.28）
Northern Mariana Islands	38.67（0-103.25）	65.88（30.9-115.18）	190.19（0-508.38）	133.79（62.85-232.92）	-1.1（-1.26,-0.89）
Norway	7639.42（2378.25-14545.31）	1193.92（452.73-2158.32）	105.58（32.75-200.91）	10.99（4.15-19.96）	-7.29（-7.51,-7.08）
Oman	5980.88（3883.84-8491.03）	8542.88（5975.66-11804.23）	852.64（560.83-1200）	428.91（298.94-592）	-2.28（-2.41,-2.17）
Pakistan	624004.04（486694.42-768360.46）	1100512.22（831179.22-1435671.77）	1123.71（872.68-1392.19）	902.25（692.83-1169.91）	-0.78（-0.87, -0.7）
Palau	16.48（0.06-44.99）	26.62（2.62-55.93）	162.89（0.66-442.41）	123.04（12.01-258.4）	-0.87（-1.11,-0.62）
Palestine	5873.11（4030.93-8300.14）	9246.36（6200.1-12441.88）	736.51（509.77-1037.99）	424.41（284.48-570.23）	-2.01（-2.31,-1.86）
Panama	5128.72（2689.37-7460.62）	3241.55（1673.03-5467.85）	344.31（180.17-500.61）	72.58（37.48-122.55）	-4.92（-5.13,-4.79）
Papua New Guinea	42703.46（30324.56-56769.42）	88671.12（62263.26-120377.96）	2353.81（1710.36-3066.39）	1736.41（1234.36-2313.52）	-0.99（-1.04,-0.95）
Paraguay	17402.78（10178.24-23706.26）	11286.52（3028.64-22854.04）	780.6（457.01-1069.4）	195.54（52.73-395.09）	-4.48（-4.74,-4.29）
Peru	79575.06（60541.49-98256.36）	57834.61（36558.88-86216.64）	633.72（486.16-783.96）	168.18（106.47-250.2）	-4.04（-4.5, -3.71）

Philippines	357397.49 (281609.55-433949.87)	700447.76（490465.35-939439.06)	1218.54 (958.92-1481.1)	832.1 (585.53-1118.26)	-1.3 (-1.43, -1.2)
Poland	368233.29 (224346.23-510487.36)	114298.2（86548.95-153258.19)	872.76 (532.83-1205.76)	158.36（119.8-211.86)	-5.53 (-5.69,-5.38)
Portugal	48547.7（18673.01-90082.29)	8221.52（4326.39-13051.52)	366.83（141.28-681.5)	30.33（16-47.92)	-7.67 (-7.94,-7.46)
Puerto Rico	966.81（0.02-3128.77)	779.45（203.69-1460.77)	27.56（0-88.96)	11.03（2.88-20.55)	-2.73 (-3.01,-2.38)
Qatar	1288.06（979.53-1625.7)	4005.47（2957.51-5200.96)	1227.17 (946.85-1541.27)	407.88 (291.55-525.56)	-3.57 (-3.84, -3.3)
Republic of Korea	238597.36（98372.54-417878)	147495.04（99160.95-205468.3)	880.23 (356.02-1532.33)	162.2（109.13-225.62)	-5.31 (-5.42,-5.22)
Republic of Moldova	55820.63 (41126.83-70078.68)	16540.07（10641.02-24365.32)	1345.82 (990.81-1692.58)	277.59 (178.74-409.59)	-5.44 (-5.66,-5.24)
Romania	268137.37 (137500.93-401353.58)	101301.48（70110.92-151903.55)	1041.34 (534.33-1554.9)	262.74 (181.64-394.36)	-4.38 (-4.5, -4.27)
Russian Federation	1353542.28 (630197.2-2164078.07)	455196.05（273650.3-707256.4)	796.35 (369.77-1270.32)	193.55 (116.42-300.29)	-4.36 (-4.63,-4.06)
Rwanda	98250.43 (75027.62-127168.72)	80640.5（56554.24-107491.2)	3417.13 (2610.19-4383.95)	1341.84 (949.81-1777.16)	-2.99 (-3.05,-2.93)
Saint Kitts and Nevis	126.52（29.79-265.33)	56.41（19.6-98.94)	345.44（81.64-724.53)	88.47（30.83-153.94)	-3.96 (-4.21,-3.65)
Saint Lucia	735.34（376.63-1180.49)	636.3（265.09-1116.99)	896.74 (459.07-1435.09)	270.37 (112.73-474.74)	-3.82 (-3.94,-3.71)
Saint Vincent and the Grenadines	501.08（284.45-793.88)	402.36（152.98-734.48)	712.96 (404.44-1129.23)	293.2（111.65-535.66)	-2.8 (-2.94,-2.69)
Samoa	1546.97（1132.88-1970.5)	1645.91（983.06-2297.6)	1846.49 (1357.7-2340.88)	1143.41 (682.03-1611.23)	-1.57 (-1.63,-1.53)
San Marino	44.31（16.23-80.9)	23.07（11.83-38.15)	121.39（44.25-220.98)	26.96（13.77-44.64)	-4.96 (-5.2, -4.8)

Sao Tome and Principe	911.15（704.25-1092.58）	1066.36（777.02-1405.17）	1445.78（1122.9-1729.25）	961.48（706.19-1243.18）	-1.32（-1.39,-1.25）
Saudi Arabia	56985.11（39155.66-79442.83）	151456.71（106773.8-198984.73）	974.03（678.78-1351.42）	706.17（513.56-904.24）	-1.12（-1.24,-1.02）
Senegal	58623.7（46590.08-72242.89）	110529.61（83420.7-143284.56）	1822.82（1454.84-2225.35）	1464.73（1109.36-1906.48）	-0.75（-0.96, -0.6）
Serbia	158720.6（87278.78-228579.73）	87405.26（61241.53-127639.49）	1704.5（952.57-2449.37）	512.13（359.73-750.21）	-3.78（-4.01,-3.63）
Seychelles	96.6（27.63-187.51）	108.79（36.25-206.66）	171.2（49.03-332.13）	95.26（31.63-179.68）	-1.91（-2.35,-1.53）
Sierra Leone	38075.13（29029.79-48010.4）	63536.19（45665.45-82835.89）	1871.26（1450.2-2327.58）	1664.38（1213.05-2138.73）	-0.39（-0.42,-0.36）
Singapore	10765.25（3848.29-18837.16）	4360.71（2289.57-6780.92）	496.59（177.56-867.29）	51.47（26.97-80.1）	-6.97（-7.29,-6.72）
Slovakia	33542.69（17269.97-53434.57）	14391.54（10439.93-18783.35）	567.45（290.78-900.13）	151.54（109.92-198.27）	-4.25（-4.37,-4.12）
Slovenia	9912.55（5137.56-15436.57）	2826.38（1991.91-3951.29）	403.42（209.09-626.67）	57.47（40.63-80.34）	-6.13（-6.3, -5.96）
Solomon Islands	3879.95（2565.9-5077.3）	8688.2（6445.22-11371.68）	2888.67（2070.73-3688.19）	2454.62（1854.97-3154.25）	-0.56（-0.61,-0.52）
Somalia	62703.82（44180.29-84672.59）	108261.71（73454.95-152822.66）	2512.85（1822.88-3294.64）	1727.45（1214.47-2405.57）	-1.17（-1.2, -1.14）
South Africa	124324.02（87106.77-165313.09）	179447.56（124697.28-241654.26）	576.08（396.14-772.42）	404.54（280.25-544.85）	-1.18（-1.31,-1.05）
South Sudan	44912.86（32060.14-59774.49）	53454.68（37116.55-74898.99）	1766.47（1270.71-2332.17）	1404.57（984.58-1930.03）	-0.73（-0.78,-0.68）
Spain	102861.97（44646.58-180968.24）	27876.03（17541.43-41500.4）	192.16（83.5-338.47）	25.57（16.13-37.79）	-6.44（-6.69,-6.12）
Sri Lanka	146120.45（120266.86-174716.54）	118899.61（58969.98-218399.52）	1508（1242.8-1790.72）	474.73（237.19-875.76）	-3.88（-4.05,-3.78）

Sudan	186928. 83 (136488. 42-245772. 73)	191774. 2 (130625. 33-263729. 54)	2006. 64 (1476. 57-2637. 56)	973. 75 (673. 56-1336. 76)	-2. 43 (-2. 5, -2. 36)
Suriname	1919. 6 (862. 97-3229. 81)	2802. 37 (1262. 78-4739. 4)	751. 13 (337. 38-1257. 41)	442. 93 (199. 95-749. 32)	-1. 67 (-1. 87, -1. 49)
Sweden	13055. 44 (3441. 07-25944. 8)	2410. 8 (695. 14-4640. 09)	81. 72 (21. 48-162. 56)	10. 03 (2. 9-19. 32)	-6. 73 (-7. 01, -6. 48)
Switzerland	15643. 58 (7269. 15-26378. 58)	3668. 77 (2275. 5-5305. 76)	141. 6 (65. 94-238. 66)	17. 89 (11. 23-25. 81)	-6. 53 (-6. 69, -6. 36)
Syrian Arab Republic	33925. 61 (22617. 53-47684. 44)	47883. 22 (30515. 68-69816. 93)	629. 53 (420. 53-883. 59)	397. 46 (259. 32-569. 85)	-1. 51 (-1. 62, -1. 37)
Taiwan (Province of China)	90009. 16 (36217. 88-169073. 02)	43643. 93 (32335. 69-56830. 47)	596. 01 (239. 45-1119. 13)	106. 32 (79. 1-138. 54)	-5. 19 (-5. 43, -4. 87)
Tajikistan	39580. 71 (28828. 24-50674. 31)	48458. 61 (33997. 64-63474. 22)	1461. 77 (1068. 6-1879. 65)	911. 51 (643. 54-1200. 7)	-1. 63 (-1. 74, -1. 49)
Thailand	401554. 21 (295417. 9-499683. 03)	386739. 71 (260662. 78-547864. 09)	1137. 13 (834. 33-1425. 56)	371. 12 (251. 58-527. 49)	-3. 56 (-3. 67, -3. 47)
Timor-Leste	5746. 13 (4398. 69-7186. 9)	11835. 27 (6602. 4-17081. 11)	2018. 68 (1547. 38-2495. 13)	1412. 25 (790. 34-2038. 07)	-1. 16 (-1. 25, -1. 1)
Togo	24032. 9 (19313. 27-29821. 44)	65376. 94 (47550. 42-86277. 62)	1926. 11 (1554. 25-2353. 43)	1725. 62 (1290. 07-2232. 79)	-0. 36 (-0. 4, -0. 32)
Tokelau	1. 97 (0. 11-6. 98)	1. 06 (0. 01-2. 7)	149. 93 (8. 7-530. 91)	72. 26 (0. 72-184. 27)	-2. 35 (-2. 48, -2. 21)
Tonga	458. 52 (331. 4-586. 18)	347. 04 (183. 99-511. 05)	852 (617. 63-1096. 01)	436. 29 (232. 01-642. 64)	-2. 23 (-2. 31, -2. 17)
Trinidad and Tobago	3632. 18 (651. 28-8323. 82)	4708 (1545. 97-8972. 85)	449. 71 (80. 34-1028. 02)	249. 9 (82. 19-475. 46)	-1. 87 (-2. 01, -1. 74)
Tunisia	20899. 8 (14002. 11-30212. 35)	32847. 02 (19980. 06-47515. 61)	458. 49 (307. 11-661. 57)	261. 16 (158. 82-377. 56)	-1. 81 (-1. 87, -1. 76)
Turkey	190164. 5 (129213. 17-264482. 37)	205514. 87 (151592. 58-267657. 25)	570. 68 (386. 78-791. 71)	231. 52 (169. 94-300. 75)	-3 (-3. 17, -2. 84)

Turkmenistan	8452.13（2213.28-17051.37）	23042.49（13145.09-37240.07）	437.6（115.16-881.27）	564.04 (321.95-910.64)	0.78 (0.48, 1.1)
Tuvalu	145.75（107.59-188.93）	38.08（21.96-57.75）	2177.81 (1614.92-2810.1)	368.85 (212.59-559.62)	-5.62 (-5.74, -5.56)
Uganda	108619.65 (81096.73-139487.39)	157670.49（111074.92-206228.17）	1732.57 (1300.88-2207.16)	1095.52 (784.09-1427.36)	-1.51 (-1.54, -1.49)
Ukraine	588109.82 (300604.2-940421.51)	195686.13（111955.3-321062.96）	851.78 (436.99-1362.29)	256.5（146.75-421.89）	-3.95 (-4.16, -3.73)
United Arab Emirates	3962.69（2801.94-5382.05）	14418.2（10013.79-19948.63）	840.4 (604.04-1132.55)	464.45 (325.45-640.78)	-1.61 (-2.4, -1.07)
United Kingdom	173343.95 (74883.05-296104.67)	38634.88（24895.82-54988.7）	187.33（80.94-319.94）	28.37（18.35-40.31）	-5.99 (-6.09, -5.89)
United Republic of Tanzania	159483.57 (125709.64-197832.41)	266987.31（198488.39-345796.24）	1489.8 (1182.39-1850.54)	1102.06 (821.95-1429.49)	-0.98 (-1.01 -0.95)
United States of America	290959.32 (115757.19-500337.6)	126711.62（62480.6-204468.71）	90.8（36.15-156.06）	22.13（10.95-35.66）	-4.52 (-4.69, -4.31)
United States Virgin Islands	57.11（1.24-154.32）	47.96（21.18-81.39）	70.15（1.54-189.98）	28.7（12.56-48.81）	-2.64 (-2.93, -2.34)
Uruguay	10810.29（4010.24-19525.37）	3970.64（1559.48-6996.69）	285.3（105.84-515.59）	70.01（27.35-123.43）	-4.45 (-4.55, -4.36)
Uzbekistan	114644.23 (72546.85-161182.59)	153014.45（104115.34-206121.73）	986.12 (623.76-1390.14)	609.09（419.8-820.85）	-1.68 (-1.97, -1.39)
Vanuatu	1689.1（1272.45-2187.11）	3608.79（2726.26-4617.19）	2576.86 (1970.84-3262.41)	1976.49 (1514.82-2504.27)	-0.88 (-0.92, -0.84)
Venezuela (Bolivarian Republic of)	27440.38 (12434.58-48124.98)	37634.71（19936.38-59637.11）	276.07（124.46-484.6）	127.84（67.7-202.85）	-2.74 (-3.04, -2.3)
Viet Nam	922582.42 (681245.03-1171417.57)	1054420.18 (679474.97-1423534.16)	2353.77 (1748.47-2984.23)	1107.14 (719.72-1485.69)	-2.46 (-2.5, -2.41)
Yemen	96490.02 (67143.19-133012.41)	154303.79（102341.97-228027.26）	2002.54 (1408.27-2757.81)	1120.01 (750.48-1651.5)	-1.99 (-2.12, -1.86)

Zambia	54315.84 (42281.26-67797.39)	107996.38 (74937.81-145966.74)	1954.18 (1530.44-2426.12)	1604.69 (1132.46-2137.25)	-0.63 (-0.66, -0.6)
Zimbabwe	39021.76 (30256.51-47578.89)	99099.33 (72657.13-130194.35)	1047.22 (813.74-1287.29)	1476.28 (1102.39-1906.67)	1.13 (1.07, 1.2)

ASMR: age-standardized mortality rate, AAPC: average annual percentage change, CI: confidence interval,SDI: sociodemographic index, UI: uncertainty interval.
